# Genome-Wide Identification and Expression Analysis of Catalase Gene Families in *Triticeae*

**DOI:** 10.3390/plants13010011

**Published:** 2023-12-19

**Authors:** Mouna Ghorbel, Ikram Zribi, Najla Haddaji, Arif Jamal Siddiqui, Nouha Bouali, Faiçal Brini

**Affiliations:** 1 Department of Biology, College of Sciences, University of Hail, P.O. Box 2440, Ha’il City 81451, Saudi Arabia; m.ghorbel@uoh.edu.sa (M.G.); n.haddaji@uoh.edu.sa (N.H.); ar.siddiqui@uoh.edu.sa (A.J.S.); n.bouali@uoh.edu.sa (N.B.); 2 Laboratory of Biotechnology and Plant Improvement, Center of Biotechnology of Sfax, P.O. Box 1177, Sfax 3018, Tunisia; ikram.zribi@cbs.rnrt.tn

**Keywords:** abiotic stress, antioxidant enzymes, gene structure, catalase, reactive oxygen species, *Triticeae*

## Abstract

Aerobic metabolism in plants results in the production of hydrogen peroxide (H_2_O_2_), a significant and comparatively stable non-radical reactive oxygen species (ROS). H_2_O_2_ is a signaling molecule that regulates particular physiological and biological processes (the cell cycle, photosynthesis, plant growth and development, and plant responses to environmental challenges) at low concentrations. Plants may experience oxidative stress and ultimately die from cell death if excess H_2_O_2_ builds up. *Triticum dicoccoides*, *Triticum urartu,* and *Triticum spelta* are different ancient wheat species that present different interesting characteristics, and their importance is becoming more and more clear. In fact, due to their interesting nutritive health, flavor, and nutritional values, as well as their resistance to different parasites, the cultivation of these species is increasingly important. Thus, it is important to understand the mechanisms of plant tolerance to different biotic and abiotic stresses by studying different stress-induced gene families such as catalases (CAT), which are important H_2_O_2_-metabolizing enzymes found in plants. Here, we identified seven *CAT*-encoding genes (*TdCATs*) in *Triticum dicoccoides*, four genes in *Triticum urartu* (*TuCATs*), and eight genes in *Triticum spelta* (*TsCATs*). The accuracy of the newly identified wheat CAT gene members in different wheat genomes is confirmed by the gene structures, phylogenetic relationships, protein domains, and subcellular location analyses discussed in this article. In fact, our analysis showed that the identified genes harbor the following two conserved domains: a catalase domain (pfam00199) and a catalase-related domain (pfam06628). Phylogenetic analyses showed that the identified wheat CAT proteins were present in an analogous form in durum wheat and bread wheat. Moreover, the identified CAT proteins were located essentially in the peroxisome, as revealed by in silico analyses. Interestingly, analyses of CAT promoters in those species revealed the presence of different cis elements related to plant development, maturation, and plant responses to different environmental stresses. According to RT-qPCR, *Triticum CAT* genes showed distinctive expression designs in the studied organs and in response to different treatments (salt, heat, cold, mannitol, and ABA). This study completed a thorough analysis of the CAT genes in *Triticeae*, which advances our knowledge of CAT genes and establishes a framework for further functional analyses of the wheat gene family.

## 1. Introduction

The formation of the fundamental parts of the ROS gene network is believed to have occurred approximately 4.1–3.5 billion years ago [1,2]. CATs, which are ROS-related proteins, are thought to have formed approximately 2.5 billion years ago. The creation of these proteins was likely vital for the survival of organisms during the Great Oxidation Event, which is estimated to have occurred approximately 2.4 to 2.0 billion years ago [1,2]. Following a significant event that altered the planet, three kinds of metalloenzymes developed in aerobic species: (i) bifunctional heme catalase–peroxidase, (ii) (non-heme) manganese CATs, and (iii) typical (monofunctional) heme CATs [3,4,5]. The latter refers to entities that have been thoroughly studied and are present in the majority of organisms [6]. CATs, or catalases (E.C, 1.11.1.6), are present in all organisms, ranging from single-celled prokaryotes to complex multicellular eukaryotes [1,2].

The production of deleterious reactive oxygen species (ROS), such as superoxide anion (O_2_^−^), hydroxyl radical (OH^−^), singlet oxygen (^1^O_2_), and hydrogen peroxide (H_2_O_2_), occurs as a result of the photosynthesis and respiration activities performed by aerobic organisms. Elevated production of reactive oxygen species (ROS) can subject cells to oxidative stress, leading to the degradation of nucleic acids, suppression of various enzymes, protein oxidation, lipid peroxidation, and ultimately cell demise [7,8]. Catalase (CAT), an antioxidant enzyme, plays a vital function in breaking down hydrogen peroxide (H_2_O_2_) into oxygen and water in order to prevent oxidative damage to cells [7]. This enzyme is mostly located in peroxisomes, although it is also present in mitochondria, chloroplasts, and the cytoplasm of cells. CAT, or catalase, plays a crucial role in plants by eliminating H_2_O_2_ generated during photorespiration, fatty acid oxidation, and mitochondrial electron transport, regardless of whether the conditions are normal or stressful [9]. In contrast to animals, plant genomes encode for distinct isozymes, with the number of isozymes varying among different species [9]. For instance, the genomes of tobacco (*Nicotiana plumbaginifolia* Viviani) and *Arabidopsis thaliana* each include three distinct genes that code for several CAT isozymes [10]. In contrast, the rice (*Oryza sativa*) genome encodes four different isozymes [11]. Furthermore, the genomes of bread wheat, durum wheat, and oats contain 10, 6, and 10 genes, respectively, that encode for CAT enzymes [12,13,14]. Furthermore, it was discovered that barley (*Hordeum vulgare*) [15] and peach (*Prunus persica*) [16] each possess two genes that encode for CAT. The tetraploid durum wheat (*T. turgidum*, AABB) and hexaploid bread wheat (*T. aestivum*, AABBDD) have acquired their A subgenomes from the wild wheat (*T. urartu*, (2n = 2x = 14; genome AuAu)) plant [17,18]. *T. urartu* has a wide range of genes, demonstrating diverse characteristics such as disease resistance, phenological properties, and morphological attributes [19]. Wild populations of *T. urartu* exhibit remarkable diversity and, due to their adaptive traits accumulated over evolutionary timescales, they have the potential to thrive in a broad spectrum of environmental conditions [20]. *Triticum urartu* serves as a valuable genetic resource for the improvement of *Tricacea* by expanding the diversity of genes in wheat. This is because certain materials derived from *T. urartu* demonstrate strong resistance against common wheat diseases such powdery mildew, stripe rust, and stem rust [21]. The affinity of *T. urartu* to the A subgenome of durum wheat and common wheat allows for the transmission of superior traits by direct hybridization and gene introgression to tetraploid and hexaploid cultivated wheat [22]. Therefore, it was deemed worthwhile to examine the composition, operation, and development of polyploid wheat genomes by conducting genomic research on *T. urartu*. The presence of wild wheat, rice, *Brachypodium*, and sorghum indicates the existence of a shared ancestor, a common grass with seven pairs of ancient chromosomes [23]. The chromosomes underwent one round of whole-genome duplication (WGD) approximately 70 million years ago, resulting in the formation of 12 pairs of ancestral chromosomes that are still present in rice. Additionally, two chromosomal fusions occurred, as indicated by previous research [24,25,26].

Through the analysis of the collinear interactions across these species, it has been determined that Tu3 and Tu6 exhibit the highest degree of conservation among the Tu chromosomes in *T. urartu*. Each chromosome, derived from a common ancestor, has the genes *Os1*, *Bd2*, *Sb3*, *Os2*, *Bd3*, and *Sb4*, in that order. Here, Os refers to *Oryza sativa*, Sb refers to *Sorghum bicolor*, and Bd refers to *Brachypodium distachyon* [27]. A comparison of the Tu3 and Ta3B chromosomes revealed variations in both protein and nucleotide composition. The syntenic sequences of Tu3 and Ta3B were determined to be 617 Mb (82.6%) and 651 Mb (84.1%), respectively. A total of 3,103 genes from Tu3, accounting for 52.32% of its genes, were successfully aligned with 3542 genes from Ta3B, representing 52.99% of its genes. This alignment was achieved by ensuring a minimum protein identity and coverage of 50%. By comparing the genes that are in the same order and position in the genomes of *Brachypodium*, rice, and sorghum, it was discovered that there are 393 deleted genes and 213 inserted genes in the collinear regions of Tu3. Similarly, in the collinear sections of Ta3B, there are 354 deleted genes and 648 added genes. Furthermore, Ta3B has been documented to undergo a recent burst of LTR retrotransposons, but Tu3 did not display this phenomenon. The increased size of Ta3B compared to Tu3 can be attributed to these changes. Furthermore, these discrepancies indicate that the amplification of LTR retrotransposons following the divergence of the A and B genomes may have influenced the evolution of the wheat genome [27]. Spelt, scientifically known as *Triticum spelta* L., is a historical sub-species of bread wheat, namely *Triticum aestivum* L. Despite being one of the older types of wheat, *T. spelta* is currently less commonly grown and is primarily planted on organic farms [28]. The remarkable health benefits, exquisite taste, and high nutritional content of this grain have sparked a renewed interest in the cultivation of *T. spelta*. *Triticum spelta* grains contain a variety of biologically active components, including dietary fiber, microelements, sterols, phenolic compounds, peptides, and vitamins [29]. Furthermore, in contrast to bread wheat and durum wheat, which necessitate rigorous agricultural methods, spelt is recognized for its ability to tolerate pathogens [29].

The wild emmer wheat, scientifically known as *Triticum dicoccoides*, with a chromosome count of 2n = 4x = 28 and a genomic composition of AABB, is an allotetraploid species. Wild emmer wheat is a species that completes its life cycle within a year. This crop is self-pollinated and has huge, elongated grains. The ears of this crop are brittle and disarticulate into spikelets when they reach maturity. The cytoplasm originates from the B genome, and the species possesses two homologous sets of chromosomes, classified as BBAA (the hybridization probably occurred spontaneously). The pollen was supplied by *Triticum urartu* Tum. ex Gandil. (AA), whereas the female function was assumed by an unnamed species strongly related to the present *Aegilops speltoides* Tausch (SS), which contributed the B genome [30]. Based on DNA evidence, the Fertile Crescent was the location where an occurrence took place approximately 360,000 years ago, leading to the evolution of the wild emmer species [31]. The process of domesticating wheat began with the cultivation of wild emmer wheat, known as *T. dicoccoides*, as evidenced by the clear historical record of wheat evolution. *Triticum dicoccoides*, commonly known as wild emmer, is naturally distributed in the Fertile Crescent region. Aaron Aaronsohn uncovered a novel finding of wild emmer wheat in its natural habitat [32]. *T. dicoccoides* was the primary crop cultivated in ancient Egypt for bread production. Presently, emmer wheat is cultivated by a limited number of traditional farming communities, primarily located in Russia and Ethiopia. The *T. durum* (durum wheat) developed from *T. dicoccoides* at a later time [33], and potentially by a separate process [34]. Understanding of the *CAT* genes in various wheat genomes remains limited, in stark contrast to the significant progress made in research on other species. In this study, a comprehensive analysis of *CAT* genes in three distinct wheat genomes (*T. dicoccoides*, *T. urartu*, and *T. spelta*) was conducted. The study focused on analyzing the quantities, positions, and compositions of individual genes, as well as their evolutionary connections. Additionally, the investigation examined the preserved sections of various proteins and their predicted subcellular locations, as well as regulatory elements in the gene promoters. These findings underscore the significant contributions of these factors to plant resistance against stress. The genomes of *T. dicoccoides*, *T. spelta*, and *T. urartu* were utilized to assess the relative expression levels of the *CAT1* and *CAT4* genes in response to various stress situations.

## 2. Results

### 2.1. Bioinformatic Analysis of CAT Genes in Triticeae Species

**Figure 1:**
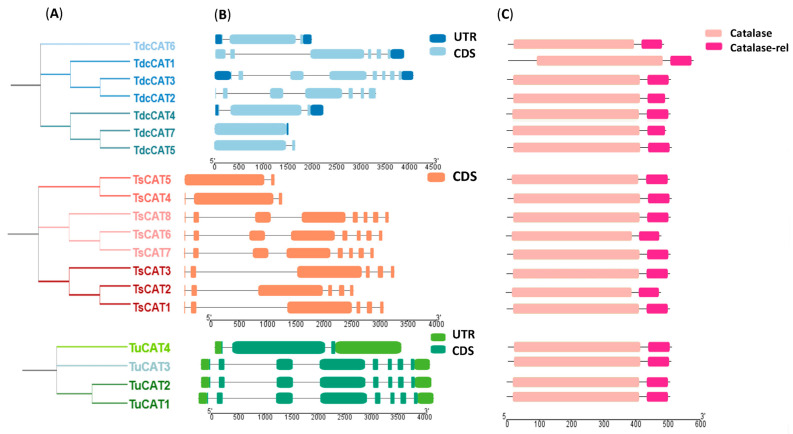
Analyses of *CAT* genes/proteins identified in *T. dicoccoide*, *T. Urartu,* and *T. spelta*: (**A**) phylogenetic analyses of CAT proteins in each species constructed by MEGA 11 and showing the phylogenetic relationship between the identified genes present in each species; (**B**) exon/intron structure of each identified gene; and (**C**) identification of conserved CAT domains (CAT-like superfamily and CAT-related superfamily) present in *Tricacea* proteins. The abscissae in (**B**,**C**) represent the lengths of the different genes/proteins. The small bows in (**B**) represent the CDS/UTR regions of the genes.

Initially, an investigation was conducted on three *Triticeae* species utilizing the Ensembl Plant database. Subsequently, the obtained data were visualized using the Tbtools software v1.123, as shown in Figure 1A. A total of 19 genes were discovered: seven genes encoding *CAT* were found in *T. dicoccoide* and designated *TdcCAT1-7*, four genes were found in the *T. urartu* genome and designated *TuCAT1-4*, and eight genes were found in the *T. spelta* genome and designated *TsCAT1-8* (Table 1). The gene numbers were arbitrarily assigned as identified in Ensembl Plant. The process of gene nomination involved selecting the first letter of the genus and species of the chosen *Triticeae*, with the exception of *T. dicoccoides*. In this case, the names (*TdcCATn*) were assigned to prevent any confusion with *TdCATs* that were identified in *T. turgidum* [13].

Subsequently, an analysis was conducted to examine protein attributes, including theoretical isoelectric point (pI) and molecular weight (MW). Table 2 displays the protein length variations in different species. In *T. dicoccoides*, protein length ranged from 494 to 559 amino acids (aa), in *T. urartu* it ranged from 492 to 494 aa, and in *T. spelta* it ranged from 487 to 494 aa. The proteins were situated on distinct chromosomes in each species. Furthermore, an examination was conducted to determine the evolutionary connections among Triticeae CAT proteins. Within each species, the CAT proteins were divided into three distinct clusters (Figure 1A). Furthermore, to comprehend the development of Tricacea genes, investigations were conducted on the exon–intron structure of *CAT* genes. Interestingly, the found *CAT* genes displayed a distinct exon/intron arrangement (Figure 1B). The number of exons ranges from one to eight in *T. dicoccoide*, from three to eight in *T. uratu*, and from two to eight in *T. spelta* (Table 1; Figure 1). Curiously, the Ensembl Plant database did not provide any information regarding the UTR region in *T. spelta* genes. In addition, the conserved domains of the putative *Triticeae* protein sequences were examined (Figure 1C). The structures of all discovered proteins consisted of catalase and catalase-related domains. Thus, it may be inferred that multiple evolutionary events, such as replication or duplication, took place over time, resulting in the differentiation of catalase genes in their structures (Figure 1B). However, they maintained their catalase identities by possessing the two conserved domains: The catalase domain (pfam00199) is a distinctive feature of catalases. Another domain, the catalase-related domain (pfam06628), has an immune-responsive amphipathic octa-peptide that is recognized by T cells in animals. However, there is currently no information available regarding this domain in plants (Figure 1C).

Finally, all identified proteins presented the same domain positions except for TdcCAT1. This protein was the longest one found, consisting of 559 aa. It also has a longer N-terminal region compared with other TdcCAT proteins. However, TdcCAT1 exhibits conserved domains that are identical to those seen in the CAT protein family of *T. dicocoides, T. urartu,* and *T. spelta* species (Figure S1).

Additional characteristics were calculated for these proteins, with molecular weights ranging from 63.8 (TdcCAT1) to 55.9 (TdcCAT2) (Table 2). Furthermore, each protein had a negative GRAVY index, signifying their hydrophilic nature. Moreover, comprehending the behavior and usefulness of proteins in different scenarios relies on evaluating protein stability. Our study revealed that 11 out of 19 discovered proteins exhibited a solitary N-glycosylation site positioned in the middle of the protein sequence. Remarkably, all the proteins that were discovered were stable, with the exception of TdcCAT1, as indicated in Table 2. Indeed, TdcCAT1 exhibited a stability score greater than 40, indicating that this protein was unstable. Unstable proteins are incapable of preserving their original structure under adverse conditions such as proteolysis, aggregation, and temperature, resulting in denaturation and loss of functionality. None of those proteins exhibited a transmembrane region, as indicated by the PROTTER database. Furthermore, the presence of four proteins (TuCAT1/2/3 and TsCAT6) with a high aliphatic index (>70) indicates that these proteins possess the ability to remain stable at various temperatures. In addition, CAT proteins exhibit a disordered area in either the N-terminal or C-terminal portions of their structures (Supplemental Table S1). Furthermore, NetPhos software (version 3.1) was employed to evaluate CAT proteins and determine the quantity of phosphorylated sites within the proteins. As anticipated, the CAT proteins that were identified exhibit distinct phosphorylation sites. The number of identified phosphorylation residues ranges from 35 to 51 (7.08–9.12%) in T. dicoccoide, which has the highest average phosphorylation level, to 35–40 residues (7.14–8.13%) in T. spelta, which has the lowest average phosphorylation level. In addition, in T. uartu, the total count of phosphorylated residues was 38 for TuCAT1/2/3 and 37 for TuCAT4, as indicated in Table S1. These findings indicate that protein phosphorylation plays a vital role in the actions of CAT proteins. 

Subsequently, the conserved domains of the discovered Triticeae protein sequences were examined. The CAT protein sequences revealed here were found to be conserved based on the multiple alignment conducted using the MEGA 11 software (Figure S2). All detected CAT proteins, regardless of their sizes, were found to possess a single CAT core domain (PF00199, catalase) and a single catalase immune-responsive domain (PF06628, catalase-related). These domains constitute the essential components of the identified CAT proteins. Furthermore, all the proteins that have been identified contain a common catalase (CAT) activity motif (CAM: FARERIPERVVHARGAS) site, site, which also includes a conserved histidine residue at Position 65 (Figure S2). Furthermore, all discovered CAT proteins have a conserved heme binding site (HBS: RVFAYGDTQ) that includes a conserved tyrosine (Y350) (Figure S2). Furthermore, the crucial amino acids H79 and Asn 159 were also preserved. Out of the 19 proteins, 11 of them contain the common PTS1-like motif (QKL/I/V). The remaining proteins have the MKV motif in their sequences. Only TdcCAT2 and TdcCAT7 do not have either of these patterns (Figure S2).

**Figure 2:**
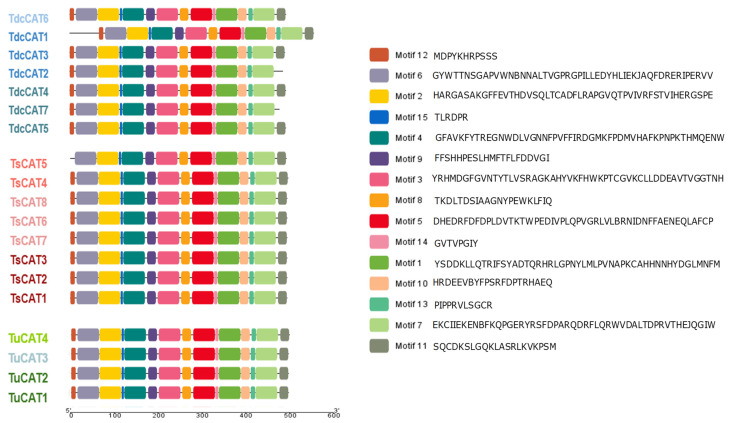
Distribution of conserved motifs in 19 identified CAT proteins in *T. dicoccoides*, *T. urartu,* and *T. spelta* recognized by the MEME search tool. Each motif is represented by a colored box. The order of the motifs corresponds to the positions of the motifs in the individual protein sequences.

The various sub-domains of the discovered CAT proteins were also examined (Figure 2). This diagram illustrates the existence of the 15 conserved motifs that have been found in the CAT proteins. In addition, two distinct proteins lacked specific motifs: TdcCAT2 did not have motif 11, while TsCAT5 did not contain motif 12.

### 2.2. Chromosomal Localization and Synteny Analyses of Tricacea CAT Genes

**Figure 3:**
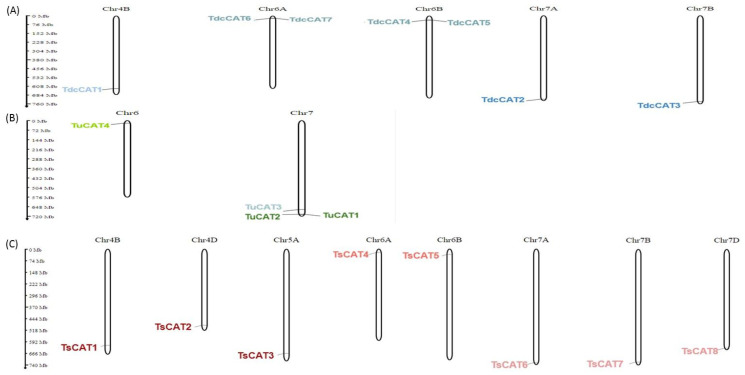
Chromosomal localisation of *TiCAT* (**A**), *TuCAT* (**B**), and (**C**) *TsCAT* genes using MG2C (v2.1). The blue color represents *TdcCAT* genes in the *T. dicoccoide* genome; the red and pink colors represent *TsCAT* genes in the *T. spelta* genome; the green color represents *TuCAT genes* in the *T. urartu* genome.

An analysis was conducted to examine the distribution of CAT genes in *Tricacea*, specifically *T. dicoccoide*, *T. urartu*, and *T. spelta*. The *CAT* genes were found on separate chromosomes in these species. The genes for *CAT* in *T. dicoccoide* are situated on chromosomes 4B, 6A, 6B, 7A, and 7B, as shown in Figure 3A. *Triticum spelta* contains a total of eight genes that encode for CAT enzymes. These genes are located on eight distinct chromosomes: 4B, 4D, 5A, 6A, 6B, 7A, 7B, and 7D (Figure 3B). In *T. urartu*, two CAT encoding genes were found on separate chromosomes, Chr6 and Chr7 (Table 1; Figure 3C). 

**Figure 4:**
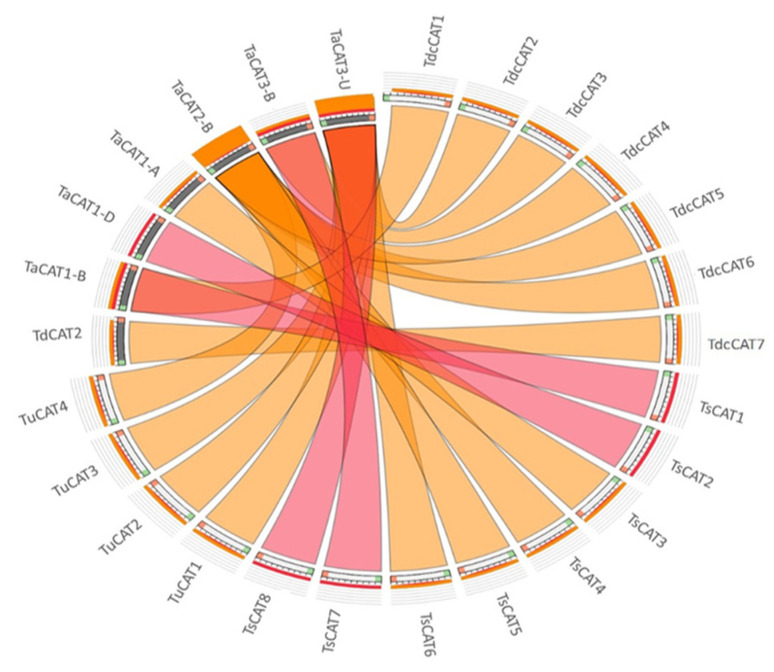
Synteny relationships of CAT proteins among the different species *T. dicocoides*, *T. urartu*, *T. spelta, T.durum,* and *T. aestivum*. Ribbons were colored based on their scores for identity, with green ≤ 75%, orange ≤ 99.9999%, and red > 99.9999%, as constructed by the Circoletto webtool.

An analysis of synteny sequence similarity was conducted to discover the evolutionary areas present across *CAT* genes from various *Triticum* species. Most TdcCAT enzymes are associated with the CAT proteins of *Triticum aestivum* (TaCAT3-U and TaCAT2-B), with the exception of TdcCAT7, which has resemblance to *Triticum durum’s* TdCAT2. Red ribbons connect two proteins that share almost 99.99% identity. Furthermore, these proteins exhibit clustering within the same group on the phylogenetic tree, specifically TsCAT7 and TaCAT3-B; TsCAT8 and TaCAT3-U; TsCAT1 and TaCAT4-B; and TsCAT2 an TaCAT1-D (Figure 4). These predictions indicate that a duplication event occurred during the evolution of the *CAT* gene in these organisms.

### 2.3. Phylogenetic Tree and Sequence Analysis of the Triticeae CAT Proteins

**Figure 5:**
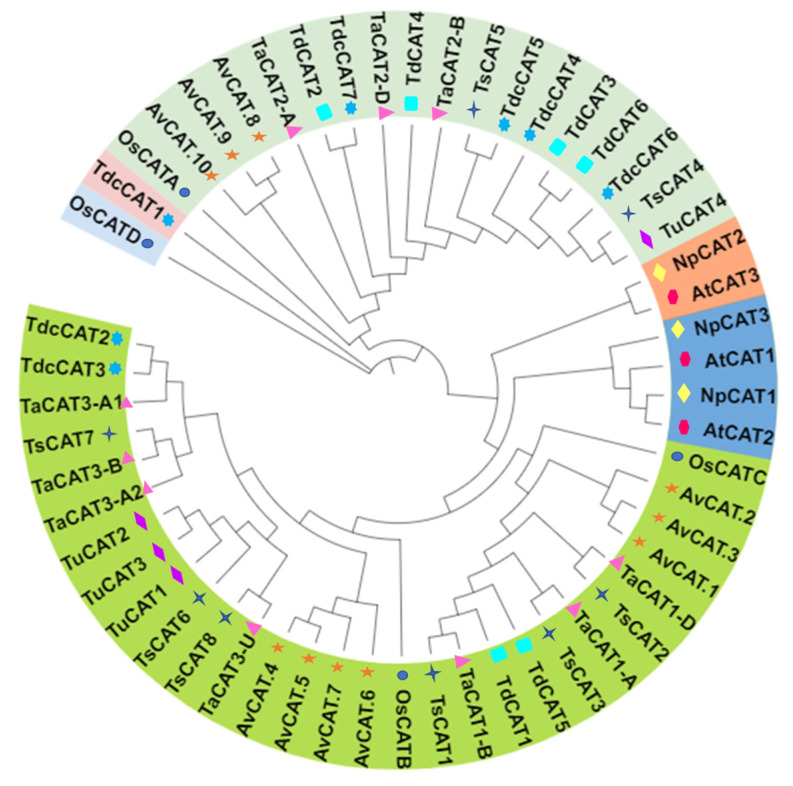
Phylogenetic analysis of CATs in bread wheat (*T. aestivum* L. Ta), durum wheat (*T. turgidum* ssp *durum* L. Td), Arabidopsis (*Arabidopsis thaliana* L. At), oat (*Avena sativa* L. Av), rice *(Oryza sativa* L. Os), tobacco (*Nicotiana plumbaginifolia* L. Np), spelta (*T. spelta* L. Ts), *T. dicoccoides* (Tdc), and *T. urartu* (Tu). The tree was generated with the full-length CAT protein sequences for bread wheat (pink triangle), durum wheat (Cyan rectabgle), *Arabidopsis* (cyan circle), oat (orange star), rice (blue circle), tobacco (yellow rhombus), spelta (blue six-pointed star), *T. dicoccoides* (Tdc), and *T. urartu* (purple rhombus). Six elementary phylogenetic groupings were displayed in the phylogenetic tree, and each group was denoted by a different background color (Scale: 0.1). The phylogenetic tree was constructed using test maximum likelihood with 1000 bootstraps using MEGA 11 software and then visualized using the iTOL web tool. The accession numbers for the CAT proteins used in this figure are: *A. sativa* L. (AVESA.00001b.r3.1Dg0003456.1; AVESA.00001b.r3.4Ag0002488.4; AVESA.00001b.r3.4Cg0001036.2; AVESA.00001b.r3.7Dg0000025.2; AVESA.00001b.r3.7Dg0002783.2; AVESA.00001b.r3.7Dg0002783.1; AVESA.00001b.r3.6Cg0000037.1; AVESA.00001b.r3.2Dg0000518.1; AVESA.00001b.r3.1Ag0002627.3; AVESA.00001b.r3.6Cg0001322.3); *T. turgidum* ssp durum (TdCAT1 WDD45561.1; TdCAT2 VAI41949.1; TdCAT3 VAI53367.1; TdCAT4 VAI53366.1; TdCAT5 VAI10245.1; TdCAT6 VAI53365.1); *O. sativa* ssp japonica (OsCATD XP_015636098.1; OsCATA: XP_015625395; OsCATC: Q10S82.1; OsCATB: XP_015643077); *A. thaliana* (AtCAT2: AAL66998.1; AtCAT1: AAQ56816.1; AtCAT3: NP_564120.1); *N. plumbaginifolia* (NpCAT1: P49315.1; NpCAT2: P49316.1; NpCAT3: P49317.1); *T. aestivum* (TaCAT1-D: TraesCS4D02G322700; TaCAT1-B: TraesCS5A02G498000; TaCAT2-A: TraesCS6A02G04170; TaCAT3-A2: TraesCS7A02G549900; TaCAT2-B: TraesCS6B02G056800; TaCAT1-A: TraesCS4B02G325800; TaCAT3-A1: TraesCS7A02G549800; TaCAT3-B: TraesCS7B02G473400; TaCAT2-D: TraesCS6D02G048300; TaCAT3-U: TraesCSU02G105300); *T. urartu:* (TuG1812G0700005868.01.T01, TuG1812G0700005870.01.T01, TuG1812G0700005318.01.T01, TuG1812G0600000378.01.T01); *T. spelta:* (TraesTSP4B01G347300.1, TraesTSP4D01G343400.1, TraesTSP5A01G526000.1, TraesTSP6A01G043000.1, TraesTSP6B01G059900.1, TraesTSP7A01G595800.1, TraesTSP7B01G508100.1, TraesTSP7D01G591500.1); and *T. Dicoccoides* (TRIDC4BG054740.1, TRIDC7AG076360.6, TRIDC7BG073240.1, TRIDC6BG007200.1, TRIDC6BG007200.2, TRIDC6AG004940.1, TRIDC6AG004940.2).

A phylogenetic analysis was conducted using MEGA 11 software (Figure 3) to investigate the relationships between CAT proteins from various species. The species included *Arabidopsis* (3 proteins), *Nicotiana plumbaginifolia* (3 proteins), *Avena sativa* (10 proteins), rice (4 proteins) and Triticeae species: *T. aestivum* (10 proteins), *T. turgidum* (6 proteins), *T. dicoccoides* (7 proteins), *T. urartu* (4 proteins), and *T. spelta* (8 proteins). The analysis utilized the entire CAT amino acid sequence from these species. The phylogenetic tree illustrates the division of 55 *CAT* genes into six classes, namely classes I-VI (Figure 5). Remarkably, the phylogenetic tree revealed that the *CAT* genes of monocot plants may be categorized into four distinct groups (Groups I-IV). Notably, the usual proteins (OsCATD and TdcCAT1) belong to a single group, indicating a unique evolutionary connection between them. Indeed, these proteins (OsCATD and TdcCAT1) undergo a distinct and separate evolutionary process, resulting in each protein being clustered in its own group. The findings indicate that the CAT proteins in Triticeae do not share any similarities with the CAT proteins in dicotyledonous species such as *Arabidopsis* and *Nicotiana plumbaginifolia*. However, there is a notable similarity between the CAT proteins in Triticeae and those in monocotyledonous plants like rice, bread wheat, durum wheat, and oat. This suggests that the evolution of CAT proteins across different species is influenced by subclasses. The evolutionary link between *Arabidopsis* and tobacco is far closer than that of monocotyledonous plants. *Arabidopsis* and tobacco proteins were classified into two distinct groups, while no monocotyledonous proteins were found in either group. On the other hand, rice and oat proteins showed a strong similarity to CAT from Triticeae, indicating that the evolutionary relationship between different species is not influenced by monocotyledonous plants, nor by the Poacea and Triticeae classes. Furthermore, upon analyzing exclusively Triticeae CAT species, we observed that the 36 Triticeae proteins could be categorized into three sub-classes. The first sub-class consisted of all CAT proteins except those from *T. urartu* (blue group), whereas the second sub-class did not include proteins from durum wheat (pink group). Ultimately, the third group consisted of proteins from all the species that were examined (Figure S3).

### 2.4. Tricacea Proteins’ Bi- and Tri-Dimensional Structures

Prediction of the secondary (2D) structure of the CAT proteins was performed. The analysis of all identified proteins indicated the existence of alpha helices, beta turns, extended strands, and random coil structures (Table 3), although no beta bridges were seen. Random coil structures constituted a significant fraction (48.17–54%) of all known CAT proteins. The proportion of proteins adopting the α-helix form ranged from 26.04% to 30.28%, whereas β-turns constituted the lowestt proportion of secondary structures, accounting for 4.47% to 6.3% of the CAT proteins. The extended strands accounted for 13.6 to 16.88% of the proteins, as seen in Table 3. 

**Figure 6:**
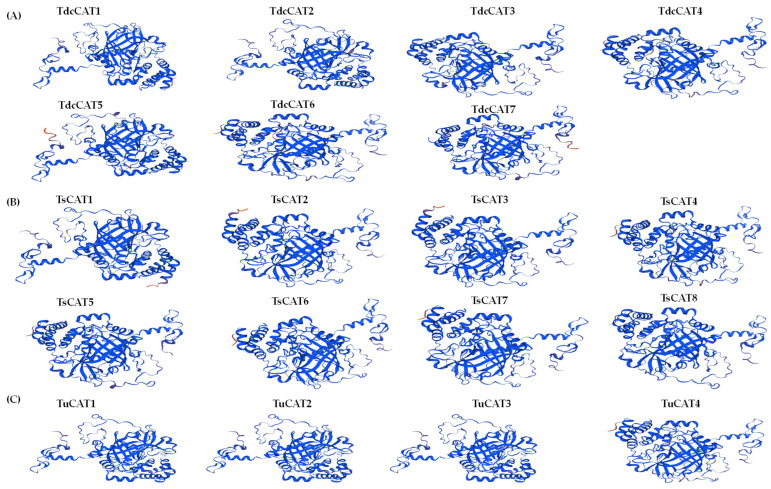
The 3D structures of CAT proteins in (**A**) *T. dicocoide*, (**B**) *T. spelta,* and (**C**) *T. urartu* built by SWISS-MODEL.

Evaluation of the 3D model’s quality was conducted using the confidence score (Figure 5). The validation parameters indicated that the model was compatible with its sequence and of excellent quality. The structures of all identified CAT proteins were mainly composed of random coils (approximately half of the protein structures) and alpha helices (concentrated in the C-terminal regions of the proteins) (Figure 6). Figure 6 demonstrates that 3D structural prediction indicates a shared homologous structure among the CAT proteins found from the putative *Triticum* species. 

### 2.5. In Silico Analysis of Tricacea CAT Proteins

**Figure 7:**
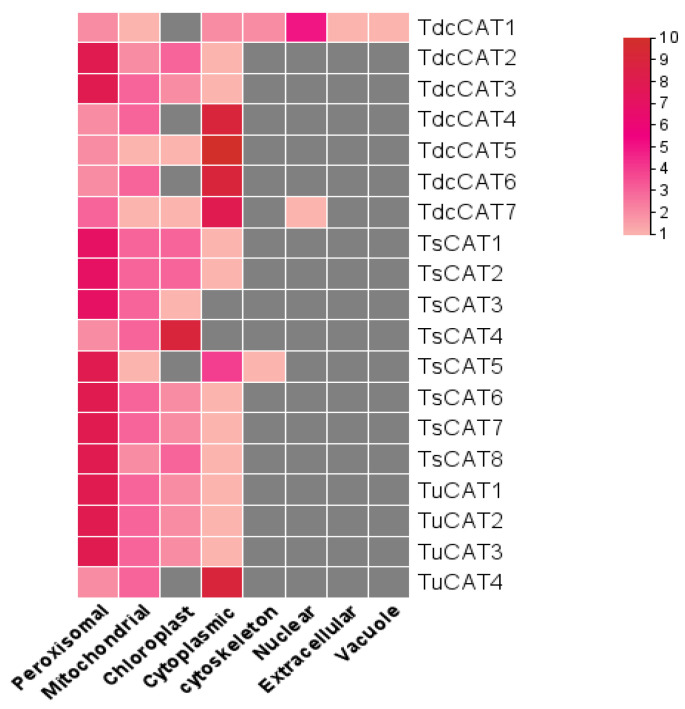
Prediction of subcellular localization of CAT proteins in *T. dicocoide*, *T. spelta*, and *T. urartu* using the Wolf PSORT online server and visualization via Tbtools software. Grey colors suggest “no prediction” of the protein in this cellular compartment.

The Wolf PSORT web server was utilized to determine the subcellular localization of Tricacea CAT proteins. The proteins that were examined exhibited distinct subcellular localizations, as depicted in Figure 6. In T. dicoccoides, two CAT proteins, TdcCAT2 and TdcCAT23, were predominantly localized in the peroxisome, while the remaining proteins were predicted to be cytoplasmic. All proteins found in *T. spelta* are located in the peroxisome, with the exception of TsCAT4, which was predicted to be located in the chloroplast. In *T. urartu*, all CAT proteins were located in the peroxisome, except for TuCAT4, which was predicted to be located in the cytoplasm (Figure 7). 

### 2.6. Identification of CaM Binding Domains

In order to determine if the identified CAT proteins contained a calmodulin binding domain, we examined the structure of these proteins using the Calmodulin Target Database. Table 4 shows that every CAT protein found contains a minimum of three potential CaMBDs situated in various regions of the proteins. All CAT proteins that have been discovered possess an IQ motif, as shown in Table 4. The biological function of IQ domains in CAT proteins is as yet uncertain, unlike the more common CaMBDs. 

### 2.7. Gene Ontology (GO) Term Distribution of Identified CAT Proteins

**Figure 8:**
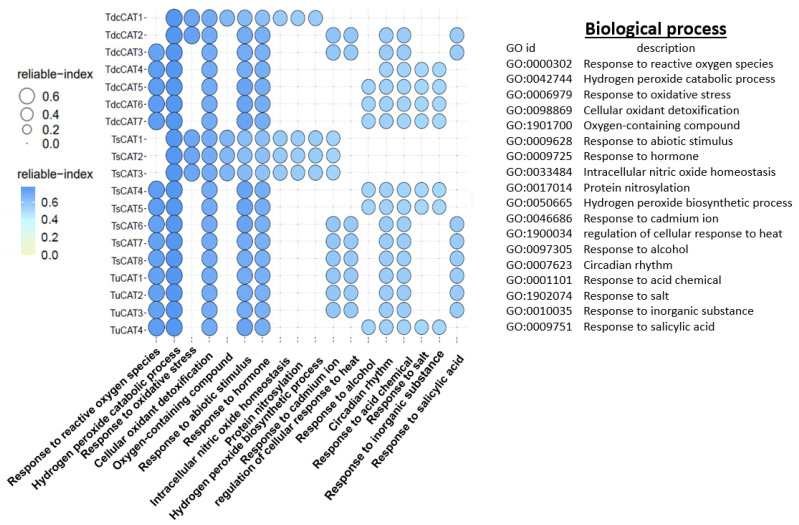
Gene ontology predictions for the TdcCAT, TsCAT, and TuCAT proteins using PANNZER and generated by SRplot webtool. The reliability of the prediction results is visualized via the intensity of the blue color and the sizes of the circles.

To identify the biological processes for the different identified proteins, we used the PANNZER2 tool. As shown in Figure 8, all identified CATs are involved in the hydrogen peroxide catabolic process, cellular oxidant detoxification, and response to abiotic stimuli and hormones. All detected catalases in *T. urartu* are engaged in responding to reactive oxygen species (ROSs), while, in other species, most catalases are involved in this process. *T. urartu*, *T. dicoccoides*, and *T. spelta* have specific CAT enzymes (TdcCAT1, TdcCAT2, TsCAT1, TsCAT2, and TsCAT3) that are involved in responding to oxidative stress. *T. urartu*, on the other hand, does not have any CAT enzymes engaged in this process. Interestingly, in *T. urartu*, CAT proteins do not participate in several processes, including intracellular nitric oxide homeostasis, protein nitrosylation, and hydrogen peroxide production. However, in *T. dicoccoides* and *T. spelta*, certain CAT proteins do fulfill these roles. In addition, various CAT proteins in each species were responsible for regulating distinct functions, including reactions to salicylic acid, cadmium, alcohol, inorganic compounds, acids, and salt. In addition, CATs also play a role in regulating the circadian cycles of proteins (Figure 8).

**Figure 9:**
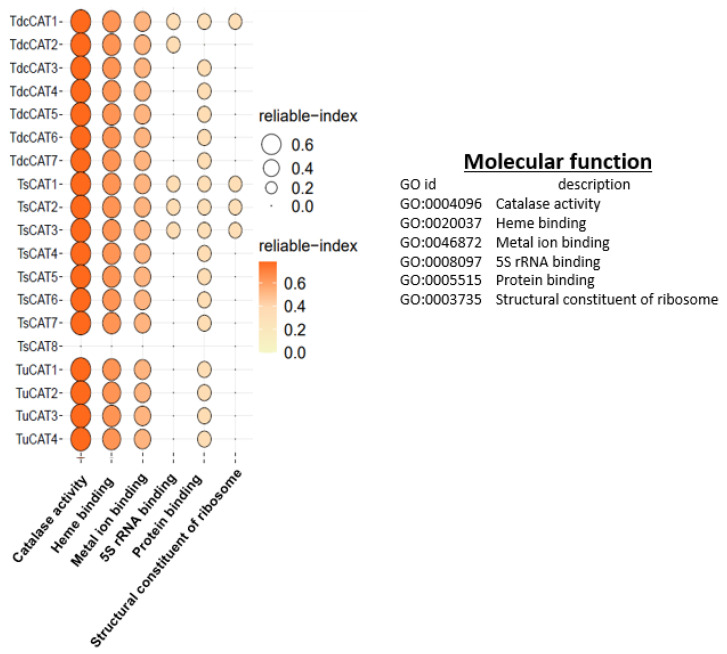
Molecular functions of TdcCAT, TsCAT, and TuCAT proteins as predicted by PANNZER and generated by the SRplot webtool. The reliability of the prediction results is visualized via the intensity of the orange color and the sizes of the circles.

To identify the molecular functions of the CAT proteins, the PANNZER2 tool was also used. The results, represented in Figure 9, show that all identified proteins have CAT activity and present heme and metal binding motifs except for TsCAT8. Interestingly, the latter protein lacks any molecular function, in contrast to the other discovered proteins, while being involved in different biological processes (Figure 8). Furthermore, within each species, certain CATs possess a 5S rRNA binding function and serve as integral components of the ribosome, with the exception of *T. urartu* (Figure 9). With the exception of TdcCAT2, all of the discovered proteins possess a protein binding function.

### 2.8. In Silico Analysis of Cis Elements

**Figure 10:**
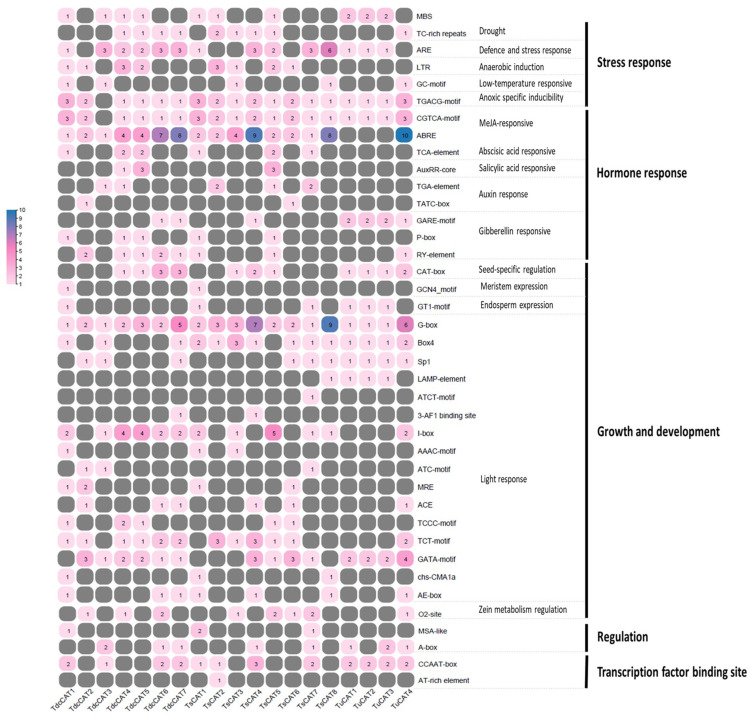
Putative cis element numbers for *TdcCAT*, *TsCAT*, and *TuCAT* gene promotors using Plantcare and visualized using Tbtools v1.123. Grey colors mean no prediction.

To examine the involvement of the *CAT* gene family in *Tricacea’s* reaction to environmental fluctuations, we conducted a thorough investigation of the cis elements present in several *CAT* genes. Initially, we utilized the PlantCARE database to identify and examine the 2000 bp region located upstream of the 19 *CAT* gene promoters that were found. This process is illustrated in Figure 10. The results of our study revealed the existence of various cis elements, including stress-responsive elements (e.g., to cold, drought, and anoxia) as well as hormone-responsive elements (e.g., to MeJA, salicylic acid, abscisic acid, auxin, and gibberellic acid). Additionally, we observed the presence of growth and development elements related to meristem expression, seed-specific regulation, light response, and cell-cycle regulation. In addition, we discovered MYB binding sites (MYBHV) responsible for drought inducibility, as well as wound-responsive components (Figure 10). 

### 2.9. Expression Analysis for CATs in Different Organs and Different Stress Conditions

**Figure 11:**
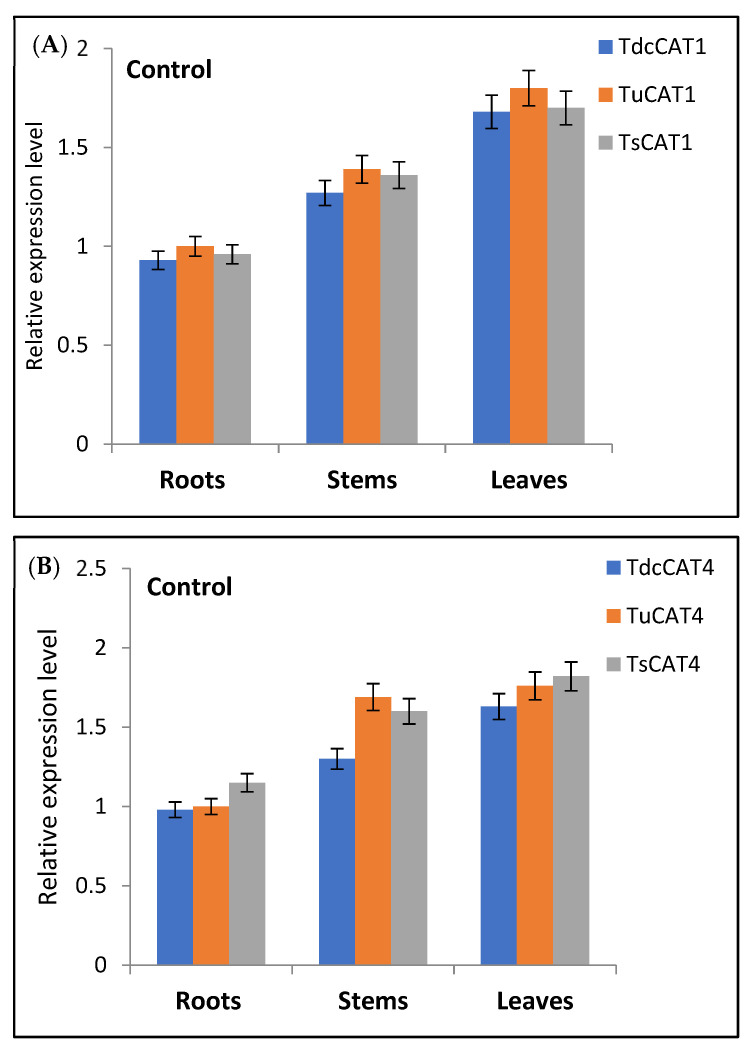
Relative expression levels of (**A**) *TdcCAT1*, *TuCAT1*, and *TsCAT1* and (**B**) *TdcCAT4*, *TuCAT4*, and *TsCAT4* in roots, stems, and leaves in normal conditions. Error bars represent standard deviation.

In normal conditions, both CAT1 and CAT4 are constitutively expressed in all tissues (roots, stems and leaves) across all three species (Figure 11). The expression level of CAT1 was marginally elevated in stems compared to roots and significantly greater in leaves across all species. 

**Figure 12:**
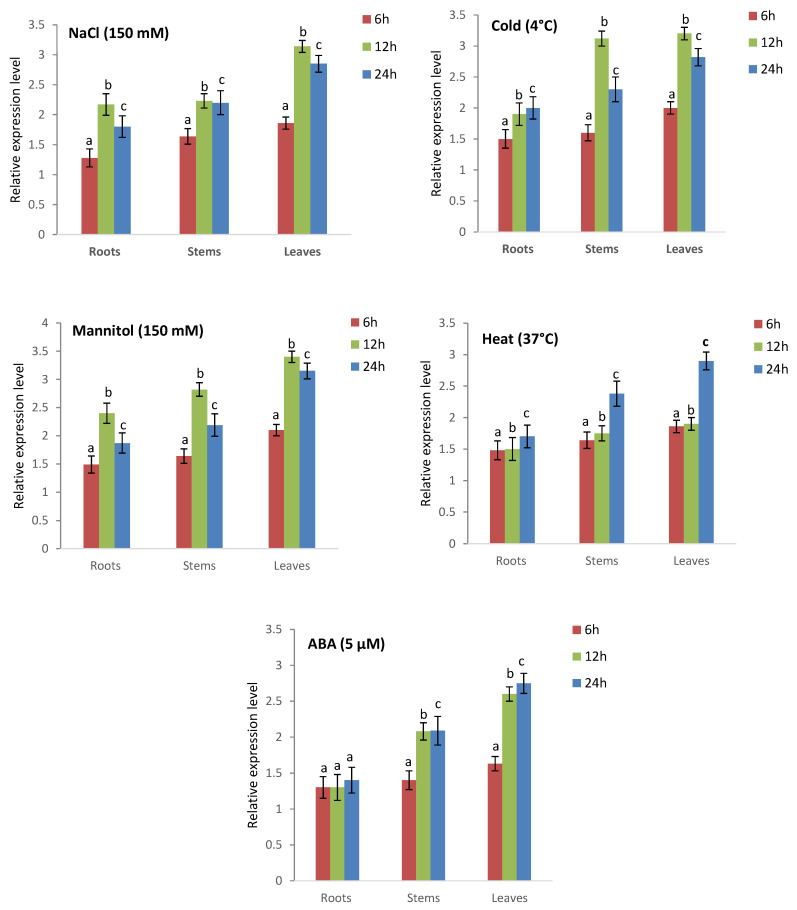
*TdcCAT1* gene expression analysis under stressful conditions. Error bars represent standard deviation (n = 15 plants). Letters indicate significant differences (two-way ANOVA test with Tukey’s pairwise comparison).

**Figure 13:**
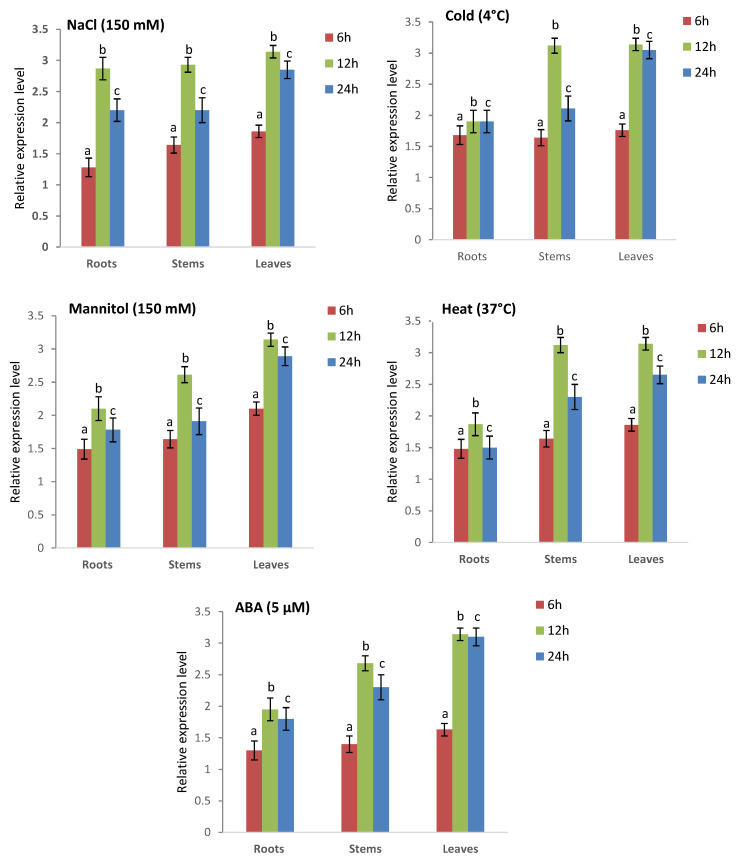
*TdcCAT4* gene expression analysis under stressful conditions. The error bars represent standard deviation (n = 15 plants). Letters indicate significant differences (two-way ANOVA test with Tukey’s pairwise comparison).

The growth and development of various *Triticum* species were impacted at the biochemical, physiological, and molecular levels under abiotic and phytohormone stress conditions. Hence, qRT-PCR was utilized to investigate the expression levels of two CAT genes (*TCAT1* and *TCAT4*) at various time intervals following exposure to NaCl (150 mM), heat (37 °C), cold (4 °C), mannitol (150 mM), and ABA (5 µM). These findings are depicted in Figure 12, Figure 13 and Figure S4–S7. Under conditions of NaCl-induced stress, the expression level of the *TdcCAT1* gene in roots showed a slight rise after 6 hours of stress. However, the expression level of this gene was more significant in leaves and stems. The highest amount of upregulation was observed in all examined tissues after 12 hours of stress exposure (Figure 12). Similar outcomes were noted in *T. urartu* and *T. spelta* (Figures S4 and S5). The expression level of the *TdcCAT4* gene exhibited an increase after 6 hours of stress administration in the investigated tissues. The *TdcCAT4* gene exhibited the lowest level of expression in roots, as compared to stems and leaves (Figure 13). The peak of this expression was observed at the 12 hour mark after stress administration, followed by a decline starting at the 24-h mark. Similar results were noted in *T. urartu* and *T. spelta* (Supplemental Figures S6 and S7).

The expressions of the *TdcCAT1* and *TdcCAT4* genes are upregulated by mannitol, cold, heat stressors, and ABA phytohormone (Figure 12 and Figure 13). The expression levels of these genes exhibited a small increase in the roots, reaching their peak after 12 hours of stress treatment in the investigated plants. Similarly, in both leaves and stems, the expression of genes was more prominently observed. An identical expression pattern was also detected in *T. urartu* and *T. spelta* (Figures S4–S7).

## 3. Discussion

Gene identification and functional classification are crucial for exploring the functions of gene families. The *CAT* gene family in plants is usually small. As an important supergene family, *CAT* has been identified at the genomic level, with availability of the whole-genome sequence in various monocotyledonous and dicotyledonous plants, such as *Saccharum spontaneum* [35], cucumber [10], *N. tabacum* [36], *E. arundinaceus* [37], and cotton [38]. However, there is limited knowledge regarding the regulatory mechanisms of these genes in relation to abiotic stressors and their involvement in governing the growth and development of three *Tricacea* plants (*T. urartu, T. dicoccoides and T. spelta*).

This study identified 19 distinct *CAT* genes from *Tricacea* plant genomes, which bear resemblance to the *CAT* genes present in durum wheat, common wheat, and oat. The genomes of *T. urartu*, *T. dicoccoides*, and *T. spelta* include four, seven, and eight genes, respectively, which encode for CAT proteins. These genes were found using the Ensembl Plant database. Remarkably, in *T. urartu*, three out of four CAT proteins (TuCAT1/2/3) exhibited identical properties, as seen in Table 2. This suggests the possibility of duplication of these proteins over time. The various CAT proteins exhibited the two conserved domains that are typical of CAT: the catalase domain (pfam00199) and the catalase-related domain (pfam06628) (Figure 1A).

Examining gene structure and conserved motifs is a vital method for comprehending the evidence of gene family evolution [39], as well as for understanding gene classification and function, in order to maintain the integrity of research. Hence, the intricate arrangement of *Tricacea CAT* genes was examined to investigate their intron–exon configuration (Figure 1B, Table 1). Genes that possess distinct intron–exon architecture and conserved domains can exhibit diverse roles [40]. It has been postulated that introns play a pivotal role in plant evolution. These components are believed to play a crucial role in enabling genes to gain new activities [41]. *CAT* genes have exon–intron architectures similar to those observed in other species, with a range of one to eight exons [13,14]. Plant *CAT* genes have been categorized into three distinct classes based on their structures and functions (photosynthetic, vascular, and reproductive) [42]. This observation was also documented at this location. The CAT proteins examined in this study were further categorized into three groups for each species (Figure 1A). An identical outcome was similarly reported for *T. durum* [13], cucumber [43], and *T. aestivum* [44], as well as for several prokaryotic and eukaryotic *CAT* genes [45]. Specifically, the number of exons in oat ranges from two to nine, while in durum wheat it ranges from three to seven [13,14]. The structure of *Gossypium hirsutum* consists of seven genes, each of which has between seven and nine exons [38].

The CAT proteins identified in this study were found to have amino acid sequences ranging from 487 to 494 aa, with the exception of TdcCAT1, which had a longer N-terminal domain of 559 aa. This unique N-terminal domain was not observed in the other identified CAT proteins, indicating that it may have distinct functions in this atypical CAT (Supplemental Figure S1). Furthermore, the GRAVY index (grand average of hydropathy) of all identified CAT proteins was found to be negative, indicating that these proteins had a hydrophilic nature. Furthermore, 11 out of the 19 discovered proteins exhibited a distinct N-glycosylation location (Table 2). Furthermore, with the exception of TdcCAT1, all proteins exhibited stability. In a recent study, researchers discovered six CAT-encoding genes in the genome of durum wheat [13]. The proteins had varying lengths ranging from 440 to 510 amino acids. All of these proteins had a distinct N-glycosylation site. Additionally, there were five, one, and five proteins that had glycosylation sites in *T. dicoccoide*, *T. urartu*, and *T. spelta*, respectively. Subsequently, an extensive investigation was carried out to analyze the evolutionary connections, conserved patterns, chromosomal positions, genetic compositions, regulatory elements, and tissue-specific gene expression patterns of several *Tricacea* plant members belonging to this gene family. This study provides comprehensive insights into the *CAT* gene family, enhancing our understanding of the roles of these genes in the plants under investigation. The absence of expected signal peptides and transmembrane regions indicates that these proteins are non-secreted rather than membrane-bound. Interestingly, none of the discovered CAT proteins had a transmembrane domain, contrary to prior findings for several CAT proteins, such as SlCAT1 in tomato [46], TtCAT1, and HvCAT1 [47]. A similar outcome was also noted in durum wheat [13], but in oat, all discovered CAT proteins lacked a transmembrane region, except for AvCAT5 [14]. The presence of a high aliphatic index in certain proteins indicates that these proteins are thermostable, as demonstrated in previous studies on TdCAT and AvCAT proteins [13,14]. Glycosylation is a common and widespread posttranslational modification in eukaryotes. It mostly occurs on the NXT/S motif of freshly synthesized polypeptides in the endoplasmic reticulum (ER) system, but can also occur in the Golgi apparatus (GA) and the secretory system. This modification regulates many signaling pathways in eukaryotes, which are believed to influence the response of plants to different stressors. Prior research has demonstrated that N-glycosylation plays a role in regulating photosynthetic efficiency through its influence over the stability of the chloroplast protein CAH1 [48,49,50]. Glycosylation regulates stomatal closure [50], photosynthesis [50], and the modulation of endogenous hormone levels [50]. The role of N-glycosylation in CAT proteins remains ambiguous and requires further exploration. However, with the exception of TdcCAT1 (Table 2), all detected proteins remained constant. Metabolic stability is essential for certain specialized cellular activities. Protein stability refers to the pace at which a protein degrades, which is measured in terms of its half-life. Proteins participate in a wide range of biological functions, hence their lifespans are influenced by several system properties that are not yet understood [51]. Protein turnover rates and half-lives, which span from minutes to years, are essential in a wide range of cellular and developmental activities. Rapid protein degradation is crucial for signal transmission, the cell cycle, and differentiation [50]. Post-translational modifications (PTMs) play a crucial role in regulating the functions of proteins involved in plant cell signaling. Phosphorylation is an extensively researched post-translational modification (PTM) that regulates various functions of proteins. This process is facilitated by several protein kinases, which, along with protein phosphatases, maintain the balance of phosphorylation in cells. This process occurs predominantly on serine (Ser) and threonine (Thr) residues in plants. It is characterized by its dynamic nature and ability to be reversed. Protein phosphorylation is responsible for regulating various cellular processes in plants. For instance, Fe homeostasis is controlled through this mechanism, as evidenced by the phosphorylation of protein [52]. In banana, fruit ripening is influenced by the phosphorylation of bZIP21, which is facilitated by mitogen-activated protein kinases 3 and 6 [53]. Similarly, the phosphorylation of NRAMP1, mediated by calcium-dependent protein kinases (CPK21 and CPK23), plays a role in maintaining manganese homeostasis in Arabidopsis [54].

Phosphorylation regulates the absorption of nitrogen, phosphorus, and potassium in plants [55]; the response of plant immunity in rice through ABA [56]; plant immunity itself [57]; the ability of Arabidopsis to tolerate drought under high-nitrogen conditions by phosphorylating NRT1.1 with CPK6 [58]; and the buildup of anthocyanins in apple fruits [59]. Phosphorylation is a type of alteration that occurs after protein synthesis and has many effects on protein function, including the activation of protein activity [60,61]. A recent study has shown that the catalase activity of durum wheat catalase (TdCAT1) was hindered following protein dephosphorylation through treatment with λ-phosphatase [61]. In addition, TdCAT1 interacts with mitogen-activated protein kinase 3 (TMPK3) through its N-terminal region and enhances its catalytic activity [62]. Our analysis in this study revealed that MAPKs can phosphorylate CAT proteins. These findings indicate that protein phosphorylation plays a vital role in the actions of CAT proteins. Additional in vivo studies are necessary to investigate the impact of phosphorylation on individual protein residues and elucidate the role of phosphorylation in various developmental processes and the plant’s response to environmental challenges.

CAT proteins typically consist of four subunits and contain two highly conserved domains: the catalase domain and the catalase-related domain. Each subunit exhibits distinct conserved sequences, including the CAT activity motif (CAM), which features a conserved histidine (FARERIPERVVH65ARGAS), heme binding sites (HBS), which contain a conserved tyrosine (RVFAY350GDTQ), and a conserved nine-amino-acid peptide (PTS1) (S/E/C-K/R/H-L) at the carboxyl terminus, which regulates the protein’s subcellular localization [63]. These traits were seen in other CAT proteins, such as CAT1 in cucumber [43], *T. monococcum*, and *T. durum* [13,64]. Furthermore, a comparison of CAT proteins from other sources revealed a significant similarity percentage (> 93%) (Figure S3), consistent with prior findings for CAT proteins found in *T. aestivum* [12], *T. durum* [13], and *N. plumbaginifolia* [65].

In addition, all discovered CAT proteins exhibited a conserved CAT activity domain, which had a characteristic histidine residue conserved at Position 65. This residue has been demonstrated to be essential for CAT activity (Figure S3). Furthermore, an additional residue that was conserved, Y350, was also detected. This residue is preserved in the heme binding site. Research has demonstrated the significance of the heme binding site in facilitating the movement of CAT into the peroxisome, indicating that proper protein folding plays a vital role in this translocation process [66]. There is a suggestion that the catalytic activity of CAT proteins relies on three specific amino acids that are conserved in discovered proteins. These amino acids include a tyrosine, which acts as the proximal ligand and coordinates the heme iron, as well as histidine and asparagine residues positioned on the opposite side. Remarkably, the distal side of the heme plane also contributes to the catalytic process [67]. The heme pyrrole IV ring and the imidazole ring of the essential catalytic His81 are almost parallel in the commonly described orientation known as the direction of His-IV. This characteristic is commonly found in CATs belonging to Clade 1. However, in CATs belonging to Clade 3, the heme group is rotated by 180°, resulting in a different orientation for the protein [68]. 

Our study revealed that *CAT*-encoding genes in *T. dicoccoide* are situated on chromosomes 4B, 6A, 6B, 7A, and 7B. Two CAT-encoding genes were found in separate chromosomes, Chr6 and Chr7, in *T. urartu*. Furthermore, in *T. spelta*, there were eight CAT expressing genes spread across eight distinct chromosomes: 4B, 4D, 5A, 6A, 6B, 7A, 7B, and 7D (Figure 3). Prior research indicated that in the durum wheat genome, six *CAT* genes were found on three distinct chromosomes (4B, 6A, and 6B). In bread wheat, however, *CAT* genes were discovered on chromosomes 4B, 4D, 5A, 6A, 6B, 6D, 7A, 7B, and an unknown chromosome (unk) [12]. Therefore, in all examined *Tricacea* species, a minimum of one *CAT* gene was assigned to chromosomes 6A and 6B (chr6 for the diploid genome of *T. urartu*). Furthermore, *CAT* genes were found to be located on chromosome 4B in all species except for *T. urartu*, and on chromosome 7 except for durum wheat. The findings indicate that *CAT* genes are conserved in chromosome 6, and to a lesser extent in chromosomes 4 and 7 in *Tricacea* species. Curiously, the *CAT* genes were exclusively found in chr5 in the hexaploid species *T. aestivum* and *T. spelta*. 

In order to investigate the collinearity relationship among CAT families in *Tricacea* species, collinearity maps were created for various wheat plants, namely *T. aestivum*, *T. durum, T. urartu*, *T. spelta*, and *T. dicoccoides* (Figure 4). Notably, the findings revealed that the majority of *TdcCAT* genes have corresponding genes in *Triticum aestivum* (TaCAT3-U and TaCAT2-B), while *TdcCAT7* has *TdCAT2* as its corresponding gene from *Triticum turgidum* ssp durum. The phylogenetic investigations corroborated these findings, since the proteins encoding genes were clustered together in the same group (Figure 4 and Figure 5). Our results offer evidence suggesting that these orthologous pairings may have existed prior to the ancient divergence of *Tricacea*.

As a further measure, the objective was to examine the correlations among CAT proteins derived from various plant species, including monocotyledonous plants such as rice, oat, durum wheat, common wheat, spelta, *T. dicoccoides*, and *T. urartu*, as well as dicotyledonous plants like *Arabidopsis* and tobacco. Our findings revealed that the 55 discovered genes were categorized into six distinct classes (class I, class II, class III, class IV, class V, and class VI) using MEGA 11 software (Figure 5). The CAT proteins were categorized into four distinct classes, while the dicotyledonous CAT proteins were classified into two groups. Our investigation revealed that several CATs belonging to distinct sub-families exhibited clustering, indicating potential occurrences of gene duplication events or convergent evolution. Remarkably, the *OsCATD* gene, responsible for producing an unconventional CAT protein found in rice, and TdcCAT1 belong to separate groups, indicating a unique evolutionary connection between them. These findings indicate that the CAT investigated does not share any similarities with the dicotyledonous species studied, but does show considerable homology with CAT from monocotyledonous species. This suggests that evolution between the various species is influenced by the subclasses. On the other hand, the study focused on identifying CAT in spelta, urartu, and dicoccoide plants. The 36 proteins were divided into three distinct subgroups, as demonstrated in Supplemental Figure S2 and previously reported [10,12]. The reliability of the group classifications in our investigation is supported by similar findings in earlier studies [10,12].

Various studies have established connections between disordered areas and important biological functions, including signaling cascades, transcription regulation, cell-cycle control, and chaperone action. The flexibility of disordered proteins enables them to interact with many patterns, exhibiting low affinity and high selectivity [68]. Therefore, the existence of these areas indicates that CAT proteins play a significant role in cellular regulation [69]. The study revealed that the CAT proteins exhibited tiny, disordered areas situated either at the N-terminal or C-terminal regions of the proteins (Supplemental Table S1). The range of disordered regions in *T. dicoccoides* is 6.09% to 22.36%, in *T. urartu* it is 6.09% to 11.94%, and in *T. spelta* it is 6.09% to 8.77%. Similar findings were previously noted in durum wheat and oat [13,14]. Indeed, TuCATs, TdcCATs, and TsCATs exhibited the lowest proportions of disordered regions when compared to durum wheat (14.5–16.25%) and oat (2.81–9.27%). Among oat proteins, only one CAT6 protein was found to lack a disordered region [14].

Protein research: The subcellular localization of proteins is a crucial biological attribute [70] that enables scientists to comprehend proteins’ biological functions. This study examined the subcellular distribution of known CAT proteins and found that most of these proteins are expected to be present in the peroxisome. However, they may also be found in the chloroplast, mitochondrion, or cytoplasm. These findings support previously published results on *T. turgidum* and *T. monococcum* (peroxisome) [64], which showed that TaCAT2A/B is present in both the cytoplasm and the nucleus [12]. Similar localization patterns were observed in rice (peroxisomes and cytoplasm) and Arabidopsis (peroxisomes) [11]. In oats, AvCATs can be found in many subcellular compartments, including peroxisomes, mitochondria, chloroplasts, and the cytoplasm [14]. These findings indicate that the location of CAT proteins in plants plays a crucial role in the removal of harmful H2O2 molecules.

The 2D protein structures were analyzed using the SOPMA servers. Remarkably, the majority of the discovered CAT proteins exhibited a prominent composition of random coils, accounting for around 50% of their overall protein structures. Similar findings were also demonstrated for durum wheat [13] and tobacco CAT proteins [71]. The proportion of random coils in the detected proteins ranged from 48.17% to 54%, while the formation of alpha helices ranged from 26.04% to 30.28%. In addition, none of the detected CATs exhibited beta bridges, as shown in other species such as durum wheat and oat [13,14]. Therefore, we can infer that CAT proteins in *Triticum* species exhibit a considerable degree of structural conservation throughout evolutionary events. In addition, the anticipated three-dimensional structures of CAT proteins from *T. dicocoide*, *T. spelta*, and *T. urartu* (Figure 6) display similar configurations, indicating that the CAT family in these species is part of a highly conserved protein group. The presence of shared conserved motifs seen in the three *Triticuim* species (Figure 2) provides significant evidence to support this deduction. We were additionally intrigued by the task of determining the biological processes associated with various catalase proteins that were found. To do this, we utilized the PANNZER2 program (Figure 8). All known CATs participate in the catabolic process for hydrogen peroxide, cellular detoxification of oxidants, response to abiotic stimuli, and response to hormones in each species. Furthermore, the four CAT proteins found in *T. urartu* are implicated in the response to reactive oxygen species (ROSs), while five CAT proteins are involved in the same biological activity in *T. spelta* and *T. dicoccoides*. This suggests the crucial role of catalase proteins in the regulation of this cellular process. The regulation of oxidative stress, intracellular nitric oxide homeostasis, protein nitrosylation, and the hydrogen peroxide biosynthesis process in *T. urartu* was not mediated by CATs, unlike in *T. dicoccoides* and *T. spelta*. As previously demonstrated in durum wheat and oat, CAT proteins play a crucial role in regulating several plant responses to environmental stressors, including salt and cadmium. 

All proteins that have been found exhibit catalase activity and possess heme and metal binding motifs, with the exception of TsCAT8 (Figure 9), which, surprisingly, lacks any molecular function but is involved in several biological processes (Figure 8). In addition, the CAT proteins that have been found exhibit various molecular roles, such as protein binding, with the exception of TdcCAT2. They also have a 5S rRNA binding function and serve as a structural component of the ribosome, except in the case of *T. urartu* (Figure 9). Additionally, the catalase proteins found in oat and durum wheat were also found to have similar activities [13,14].

Calmodulins (CaMs) are extensively researched calcium sensors [72,73,74]. These proteins are characterized by their small size, acidic nature, and high degree of conservation among eukaryotes. They play a crucial role in detecting minor fluctuations in intracellular Ca^2+^ levels. This, in turn, allows them to regulate plant responses to different growth processes and adapt to varying environmental situations. Calmodulins have the capability to bind with numerous ligands, including MAP kinase phosphatase [75], transcription factors [76], pathogen-related protein (PR-1) [77], and catalase [78,79,80,81]. This study demonstrates that the CAT proteins found possess a varying number of domains, ranging from five to three, with each protein including one IQ motif. These findings are summarized in Table 4. In durum wheat and oat, it was also found that CAT proteins contain at least three CaMBDs, which are positioned at separate parts of the proteins and have just one IQ motif [13,14]. The interaction between TdCAT1 and CaM, which is not dependent on Ca2+, improves the catalytic activity of TdCAT1 in a manner that is dependent on the presence of Calcium [81]. Furthermore, studies have demonstrated that the catalytic function of CAT1 proteins in Arabidopsis and sweet potato was augmented when the Ca^2+^/CaM combination was present [79,80,81].

Additionally, a gene ontology study was conducted. The results verified that all discovered proteins exhibited catalase activity and possessed heme/metal binding motifs, indicating the significance of heme and other cations in the catalytic activity of the proteins. Recent evidence has shown that the stimulation of TdCAT1 occurs in the presence of Fe^2+^ and other cations such as Mn^2+^, Mg^2+^, Ca^2+^, Zn^2+^, and Cu^2+^, but not in the presence of Cd^2+^ [81]. 

*Tricacea* catalases have distinct functionalities. All known CATs regulate the cellular process of oxidant detoxification, the breakdown of oxygen-containing compounds, and the catabolic process of hydrogen peroxide. These proteins play a role in the cellular response to reactive oxygen species (ROS) and are also engaged in responding to hormones and various abiotic stressors. Additionally, they are implicated in the process of protein nitrosylation. Certain proteins play a role in the plant’s reaction to heat and alcohol, and also regulate the plant’s response to cadmium, as demonstrated in previous studies on *T. aestivum* [12] and *T. durum* [13]. These studies indicate that CATs may have significant functions in the plant’s response to different stressors and have supplied valuable information about the *CAT* family genes in distinct *Tricacea* genomes. 

Recently, cis-acting elements were discovered in *T. aestivum* [12] and *T. durum* [13], but no investigation has been conducted on *T. urartu*, *T. spelta*, or *T. dicoccoides*. Our study identified various stress-sensitive components, including hormone-responsive elements (ABA, SA, and auxin), drought- and light-responsive elements, and cell-cycle regulation elements. Similar regulatory components were previously identified in *T. aestivum* [12] and *T. durum* [13]. The aforementioned findings indicate that the identified *CAT* genes are likely to have a role in plant development and growth, and are involved in cell differentiation as regulators of reactive oxygen species (ROS). 

CAT plays a crucial role in oxidative senescence, growth, and development, and serves as a defense mechanism against environmental stress in plants. Catalase (CAT) activity can be influenced by various factors, including heavy metals, light exposure, salt concentration, temperature, drought conditions, plant hormones, and infections [11,12,13,14,15,16]. Prior studies have demonstrated variation in the expression patterns of the *CAT* gene in plants in response to diverse environmental circumstances [12,13,14]. The initial *CAT* gene (TdCAT1) obtained from the durum wheat genome has significant expression across the entire plant during all stages of development in durum wheat (*T. turgidum* ssp. durum) [82]. *T. monococcum* exhibited comparable outcomes [64]. The expression of *AvCAT2*, *AvCAT4*, and *AvCAT8* genes was examined in oat to assess their response under different stress conditions. The genes exhibited a constant expression in all examined tissues, including roots, stems, and leaves. *AvCAT2* showed no alterations in gene expression or transcription level when exposed to heat stress at 37 °C [14]. Remarkably, the expression of the *AvCAT4* and *AvCAT8* genes exhibited a rapid surge within a mere hour of stress exposure, reached its highest point after 12 hours, and subsequently began to fall after 24 hours [14]. Furthermore, it was observed that only AvCAT4 exhibited an increase in expression levels in plants subjected to cold stress, indicating the significant role of AvCAT4 in oat’s ability to respond to high temperatures. The genome of *Nicotiana tabacum* L. has seven distinct *CAT*-encoding genes, which have been categorized into three separate groups [71]. This classification has also been observed in other species [12,13,14]. Evidence indicates that *NtCAT1-4* had robust expression in shoots, while *NtCAT5* and *NtCAT6* displayed abundant expression in roots. In addition, *NtCAT7* controlled circadian rhythms. Notably, the expression pattern of *NtCATs* was significantly affected by drought stress. In addition, the expression of *NtCAT5/6/7* increased in response to cold stress, but decreased in response to drought and salt stress [71]. The results of our study have revealed fresh opportunities for future research and provided insights into *CAT* family genes across several wheat species. In this study, we examined the transcript levels of two *CAT* genes from distinct subgroups (*CAT1* and *CAT4*) in each species under investigation. The genes were examined in various conditions, including high and low temperatures; mannitol, NaCl, and ABA treatment; and three distinct tissues: roots, stems, and leaves. This analysis is depicted in Figure 12 and Figure 13. The genes exhibited a constitutive expression pattern under normal conditions, with the roots showing the lowest level of expression for both genes across all species examined. The results of our study indicate that both genes were elevated in the species we analyzed. The level of upregulation was more significant in leaves compared to roots and stems. This finding is supported by Figure 11 and Figure 12. The greatest induction for both *CAT1* and *CAT4* genes was observed after 12 hours for all tested conditions. This suggests that these genes respond quickly to ensure the detoxification of reactive oxygen species (ROS) and reduce the effects of oxidative stress. This is supported by Figure 11 and Figure 12, as well as Supplemental Figures S4–S7. These findings indicate that *CAT1* and *CAT4* genes in the examined *Triticum* species are vital for plant defense against various environmental circumstances and are also involved in various growth and development processes, as evidenced by their consistent expression. *Further* research is required to comprehensively comprehend the functionalities of the *Tricacea* CAT genes. The results of our study provide insight into *Tricacea CAT* family genes and open up new possibilities for future investigations. 

## 4. Materials and Methods

### 4.1. Plant Material

*T. dicoccoides* (*cv.* IC 9132), *T. urartu* (*cv*. Sharka), *and T. spelta* (*cv.* Caeruleum) seeds were obtained from ICARDA, Syria and used in this study. Before incubation, 20 mL of a 0.5% sodium hypochlorite solution was applied to nearly 40 seeds and left on for 15 min. After that, 40 mL of sterile water was used to wash the seeds five times to eliminate the remaining sodium hypochlorite. Seed incubation was carried out at 25 °C for 16/8 h under light/dark conditions and 280 mol m^2^ s^1^ of photosynthetically active radiation. Petri dishes with a sheet of Whatman filter paper were used to germinate seeds. Seeds were then placed in a greenhouse.

Ten days after incubation, several stress treatments were applied to the seedlings. The stress treatments employed in this study included distilled water as a control, 200 mM mannitol, 150 mM NaCl, cold stress (4 °C), heat stress (37 °C), and 5 mM ABA. All treatments were conducted for 24 h. Each therapy was carried out three times. Instantly after being harvested, the shoots and roots were frozen in liquid nitrogen and preserved at −80 °C.

### 4.2. Identification of Triticum CAT Gene Families

The specific conserved domains of catalase PF00199 and pfam06628 were used as a query to run Blastp in the genomes of *Triticum* species. These sequences were verified using the Ensembl Plant database (https://plants.ensembl.org/index.html; accessed on 8 June 2023). The obtained catalase proteins were scanned by interpro (https://www.ebi.ac.uk/interpro/; accessed on 10 June 2023) [83], CD-Search (https://www.ncbi.nlm.nih.gov/Structure/cdd/wrpsb.cgi/; accessed on 13 June 2023) [84]; and HMMER (https://www.ebi.ac.uk/Tools/hmmer/; accessed on 13 June 2023) [85]. Thus, 19 CAT proteins were obtained and selected for further studies.

### 4.3. Characterization of Tricacea CAT Proteins and Genes

The ProtParam program on the ExPASy website was used to determine the CAT proteins’ physical and chemical characteristics, including their amino acid count, molecular weight (MW), isoelectric point (pI), hydrophobicity and instability index (https://web.expasy.org/protparam/; accessed on 19 June 2023). The web program Wolf PSORT (https://wolfpsort.hgc.jp/; accessed on 22 June 2023) [86] was used to predict the subcellular localization of different identified CAT proteins. The MEME webtool (https://meme-suite.org/meme/; accessed on 26 June 2023) [87] was used to examine conserved motifs with the following criteria: the maximum number of motifs was 15, the ideal width was set between 10 and 50, and there were either zero or one occurrences of each motif per sequence. The Pfam protein family database (Pfam 35.0; http://pfam.xfam.org/, accessed on 26 June 2023) was used to identify motifs that corresponded to the pfam domain [88].

Gene ontology (GO) analysis was used to predict molecular function and biological processes using PANNZER2 [89] (http://ekhidna2.biocenter.helsinki.fi/sanspanz/; accessed on 23 June 2023); the results were visualized using the SRplot online tool (http://www.bioinformatics.com.cn/srplot/; accessed on 23 June 2023) [90].

### 4.4. Gene Structure and Conserved Motifs of CAT Genes

General Feature Format (GFF) 3 files were downloaded from the Ensembl Plant database and used in the Tbtools software to visualize intron/exon gene organizations. The Protter database (https://wlab.ethz.ch/protter/start/, accessed on 24 July 2023) was used to study the presence of transmembrane domains and signal peptides in the identified CAT gene structures. Finally, the search for putative CaMBDs was carried out using the Calmodulin Target Database (http://calcium.uhnres.utoronto.ca/ctdb/no_flash.htm [91], accessed on 26 June 2023).

### 4.5. Chromosome Location and Phylogenetic Analysis of the Tricacea Gene Family

Based on Ensembl Plant information regarding the location, the CAT gene families identified in the selected *Triticum* species were discovered. The MG2C server was used to create the chromosomal location map (http://mg2c.iask.in/mg2c_v2.1/; accessed on 23 July 2023) [92]. Protein multiple-amino-acid sequence alignment (MSA) was performed with the cluster W algorithm using MEGA 11 [93]. The phylogenetic tree was generated with the use of the maximum likelihood method with 1000 bootstraps and visualized with the iTOL v6.8 web tool (https://itol.embl.de/upload.cgi; accessed on 22 July 2023) [94].

### 4.6. Evolutionary Relationship of the Catalase Sequences

Syntenic relationships among *T. dicocoides*, *T. urartu*, *and T. spelta* protein catalases and their orthologues in *Avena sativa*, *T. durum,* and *T. aestivum* (which were used as queries) were analyzed using the Circoletto webtool (https://bat.infspire.org/tools/circoletto/; accessed on 3 August 2023) [95].

### 4.7. The 2D and 3D Structures of Identified Catalase Proteins

The SOPMA server (https://npsa-prabi.ibcp.fr/cgibin/npsa_automat.pl?page=npsa_sopma.html; accessed on 18 July 2023) was used to anticipate the 2D structures of the identified CAT proteins, whereas 3D structures were predicted using the SWISS-MODEL server (https://swissmodel.expasy.org/; accessed on 18 July 2023).

### 4.8. Promoter Cis-Regulatory Element Analysis of the CAT Gene Family

A 2 kb sequence upstream of the translation start site of selected *Tricacea* CAT genes was retrieved from the corresponding genome as the promoter sequence (obtained from Ensembl Plant (https://plants.ensembl.org/index.html; accessed on 2 August 2023) and its cis-regulatory elements were predicted using PlantCARE (http://bioinformatics.psb.ugent.be/webtools/plantcare/html/; accessed on 4 August 2023) [96]. The results were visualized using TBtools [90].

### 4.9. RNA Extraction and Quantitative Real-Time Reverse Transcription PCR (QRT-PCR)

To extract the total RNA, the RNeasy Plant Mini Kit (QIAGEN, Hilden, Germany) was used. RNA extraction was performed from the roots, shoots, and leaves of the investigated *Tricacea* plants independently (0.5 g of each tissue). After extraction, RNA was separated from genomic DNA using an RNase-free DNase kit (QIAGEN), measured, and then used to synthesize first-strand cDNA with an oligo-dT primer from Promega’s GoScript Reverse Transcription System (Madison, WI, USA). cDNA (obtained from 40 ng of RNA that had undergone DNase treatment) at an amount of 2 µL; each primer for the *CAT* genes (Table S2) at an amount of 0.5 µL and a concentration of 10 µM; 5 µL of 2 × SYBR Green I master mix; and 1 µL of RNase-free water were used for the PCR reactions, which were carried out at a final volume of 10 µL. A denaturation step at 95 °C for 5 min, 40 cycles of 10 s at 95 °C, 20 s at 60 °C, and 30 s at 72 °C and a melting curve made up of 5 s at 95 °C, 1 min at 65 °C, and 5 min of an increase in temperature from 65 °C to 97 °C made up the reactions. Each sample received three technical repetitions and three biological repetitions for each stress state. Melting curve analysis was used to determine whether there had been a single amplification after the cycling process. At the end of the experiment, the triplicate PCR threshold cycle (CT) data were averaged and used for transcript quantification.

Using the *α-tubilin* and the *actin* genes created from the *T. aestivum* genome (Table S2) as an internal expression standard, the relative expression ratio of the *Tricacea* CAT genes was computed (Table S2) [97]. Based on triplicate data, the relative expression level was determined using the 2^−∆∆CT^ formula, where ∆∆CT = (CT, target gene CT, tubilin) stressed (CT, target gene CT, tubilin). Three separate experiments (three biological replicates) with varying relative expression ratios are given.

### 4.10. Statistical Analysis

All analyses were performed in three replicates. Statistical analyses were carried out with the aid of GraphPadPrism9. A two-way ANOVA was used for each factor (plant part, species, time after stress application) separately in order to compare the differences between treated and non-treated plants, and Tukey’s pairwise comparison tests were conducted afterwards (data were following a normal distribution), with a significance level of α = 0.05 in relation to the control group of untreated plants.

## 5. Conclusions

Catalase proteins play a vital role in preventing cell death by transforming the hazardous substance H_2_O_2_ into safe components. The identification, isolation, and molecular characterization of several wheat (*T. urartu*, *T. spelta*, and *T. dicoccoides*) genes remain unknown, despite the crucial role of catalase genes in plant defense against diverse abiotic stress conditions. In this study, we examined different computational analysis methods to enhance our comprehension of the CAT family in the chosen wheat plants as a collective entity. Through analysis of the genomes of the three species, we have identified a total of seven catalase-encoding genes (*TdCATs*) in *T. dicoccoides*, four genes in *T. urartu* (*TuCATs*), and eight genes in *T. spelta* (*TsCATs*). These genes possess the two conserved domain characteristics of catalases, namely pfam00199 and pfam06628. The catalase gene families in all of these animals were divided into three subfamilies. Each species had these genes distributed across distinct chromosomes. Various bioinformatic studies have demonstrated that the identified catalase proteins exhibit a high degree of structural conservation, including heme binding domains, cation binding domains, catalase activity motifs, peroxisomal targeting signal 1 (PTS1-like) domains, calmodulin binding domains, and catalase-related motifs. Additional bioinformatic analysis has shown that the structures of several catalase proteins that have been found are significantly conserved. Furthermore, analysis of the *CAT* gene promoters unveiled many cis elements located in the upstream area of the *CAT* genes. These constituents were detected in durum wheat and bread. The expression of genes related to growth, development, hormone response, and stress may be influenced by wheat and corn. 

## Figures and Tables

**Table 1:** List of the predicted CAT proteins identified in *Triticeae* species: the putative identified CAT proteins are listed. Gene length, protein length, chromosome location, and number of exons were analyzed.

	Transcript ID	Chr	Strand	Length of Gene (bp)	Length of Protein (aa)	Star…End	N° of Exon
*TdcCAT1*	TRIDC4BG054740.1	4B	reverse	1971	559	622.471637 --- 622.475660	6
*TdcCAT2*	TRIDC7AG076360.6	7A	forward	1469	487	716.334208 --- 716.336654	7
*TdcCAT3*	TRIDC7BG073240.1	7B	forward	2056	492	735.491351 --- 735.493975	8
*TdcCAT4*	TRIDC6BG007200.1	6B	reverse	1805	494	36.233928 --- 36.236074	3
*TdcCAT5*	TRIDC6BG007200.2	6B	reverse	1485	494	36.234183 --- 36.235788	2
*TdcCAT6*	TRIDC6AG004940.1	6A	forward	1809	494	19.346843 --- 19.348894	3
*TdcCAT7*	TRIDC6AG004940.2	6A	forward	1476	479	19.347133 --- 19.348608	1
*TuCAT1*	TuG1812G0700005868.01.T01	7	reverse	1888	492	705.363621 --- 705.367539	8
*TuCAT2*	TuG1812G0700005870.01.T01	7	reverse	1907	492	705.405522 --- 705.409459	8
*TuCAT3*	TuG1812G0700005318.01.T01	7	reverse	1888	492	669.038070 --- 669.041988	8
*TuCAT4*	TuG1812G0600000378.01.T01	6	forward	2592	494	19.198632 --- 19201466	3
*TsCAT1*	TraesTSP4B01G347300.1	4B	reverse	1479	492	612.241373 --- 612.244903	6
*TsCAT2*	TraesTSP4D01G343400.1	4D	reverse	1479	492	483.842484 --- 483.845478	6
*TsCAT3*	TraesTSP5A01G526000.1	5A	reverse	1479	492	664.922332 --- 664.926051	6
*TsCAT4*	TraesTSP6A01G043000.1	6A	forward	1485	494	22.240310 --- 22.242037	3
*TsCAT5*	TraesTSP6B01G059900.1	6B	reverse	1473	490	35.462733 --- 35.464326	2
*TsCAT6*	TraesTSP7A01G595800.1	7A	forward	1479	492	725.034291 --- 725.037799	8
*TsCAT7*	TraesTSP7B01G508100.1	7B	Forward	1479	492	716.979044 --- 716.982402	8
*TsCAT8*	TraesTSP7D01G591500.1	7D	Forward	1479	492	628.479984 --- 628.483599	8

**Table 2:** Physico-chemical characteristics of identified catalase proteins in *Tricacea*.

		Number of aa	Molecular Weight (MW)	Isoelectric Point (pI)	GRAVY Index	N-Glycosylation Site	Instability Index	Alipahtic Index	Number of Asp and Glu Residues	Number of Arg and Lys Residues
*T. dicoccoide*	TdcCAT1	558	63,897.91	8.23	−0.643	N314	44.02 Unstable	66.82	68	70
	TdcCAT2	487	55,998.16	6.26	−0.490	--	32.62 Stable	69.10	61	52
	TdcCAT3	492	56,677.13	6.49	−0.525	--	31.50 Stable	68.82	62	56
	TdcCAT4	494	56,972.36	6.50	−0.536	N248	32.72 Stable	65.94	68	63
	TdcCAT5	494	56,918.24	6.53	−0.524	N248	32.51 Stable	67.11	67	62
	TdcCAT6	494	56,979.35	6.47	−0.542	N248	32.89 Stable	65.74	68	63
	TdcCAT7	494	55,251.16	6.24	−0.531	N248	31.57 Stable	66.98	66	58
*T. urartu*	TuCAT1	492	56,489.94	6.56	−0.505	--	30.54 Stable	70.14	61	56
	TuCAT2	492	56,489.94	6.56	−0.505	--	30.54 Stable	70.14	61	56
	TuCAT3	492	56,489.94	6.56	−0.505	--	30.54 Stable	70.14	61	56
	TuCAT4	494	56,874.28	6.58	−0.532	N247	32.94 Stable	66.66	67	63
*T. spelta*	TsCAT1	492	56,793.96	6.52	−0.595	N247	37.60 Stable	69.15	63	58
	TsCAT2	492	56,737.90	6.54	−0.593	N247	36.94 Stable	69.35	63	58
	TsCAT3	492	56,709.85	6.54	−0.593	N247	36.26 Stable	68.94	63	58
	TsCAT4	494	56,874.28	6.58	−0.532	N247	32.94 Stable	66.66	67	63
	TsCAT5	490	56,420.71	6.62	−0.528	N243	32.95 Stable	67.41	66	62
	TsCAT6	492	56,516.02	6.56	−0.496	--	30.54 Stable	70.93	61	56
	TsCAT7	492	56,534.05	6.78	−0.517	--	30.59 Stable	69.35	60	57
	TsCAT8	492	56,549.99	6.65	−0.515	--	30.80 Stable	69.64	61	65

**Table 3:** The 2D structural composition of *Tricacea* CAT proteins as revealed by the SOPMA online software (https://npsa-prabi.ibcp.fr/cgi-bin/npsa_automat.pl?page=/NPSA/npsa_sopma.html, accessed on 2 August 2023). Alpha helix (Hh)/Extended strand (Ee)/Beta turn (Tt)/Random coil (Cc)/. Beta bridges were absent in all identified proteins.

		Alpha Helix (Hh%)	Extended Strand Ee (%)	Beta Turn Tt (%)	Random Coil Cc (%)
*T. dicoccoide*	TdcCAT1	26.12	13.6	5.9	54.39
	TdcCAT2	27.25	15.57	5.94	51.23
	TdcCAT3	29.1	14.81	4.87	51.32
	TdcCAT4	26.26	14.34	6.06	53.33
	TdcCAT5	26.87	16.16	5.25	51.72
	TdcCAT6	26.46	15.15	5.66	52.73
	TdcCAT7	26.04	16.88	6.25	50.83
*T. urartu*	TuCAT1	30.08	15.24	5.08	49.59
	TuCAT2	28.25	15.45	5.69	50.61
	TuCAT3	28.86	15.24	5.49	50.41
	TuCAT4	28.25	15.24	6.3	50.20
*T. spelta*	TsCAT1	26.63	16.26	5.28	51.83
	TsCAT2	30.28	15.45	6.10	48.17
	TsCAT3	27.03	16.26	5.83	50.81
	TsCAT4	25.91	15.79	5.26	53.04
	TsCAT5	26.33	14.69	5.1	53.82
	TsCAT6	28.05	15.45	5.28	51.22
	TsCAT7	27.64	15.45	5.49	51.42
	TsCAT8	27.03	15.45	4.47	52.64

**Table 4:** Number of identified CaMBDs in *Tricacea* CAT proteins. The number of CaMBDs was identified using the Calmodulin Target Fatabase. The number of identified domains varies from three to five.

		Protein Length	Number of Putative CaMBDs	Typical CaMBD	Position	IQ Motif	Position
*T. dicoccoide*	TdcCAT1	559	5	4	24–47; 125–146; 275–296; 529–553	1	363–382
	TdcCAT2	487	3	2	58–79; 204–224	1	296–315
	TdcCAT3	492	3	2	58–79; 204–224	1	297–315
	TdcCAT4	494	4	3	58–79; 207–229; 341–360	1	296–315
	TdcCAT5	494	4	3	58–79; 207–229; 341–360	1	296–315
	TdcCAT6	494	4	3	58–79; 207–229; 341–360	1	296–315
	TdcCAT7	494	4	3	58–79; 207–229; 341–360	1	296–315
*T. urartu*	TuCAT1	492	3	2	58–79; 204–224	1	296–315
	TuCAT2	492	3	2	58–79; 204–224	1	296–315
	TuCAT3	492	3	2	58–79; 204–224	1	296–315
	TuCAT4	494	4	3	58–79; 207–229; 341–360	1	296–315
*T. spelta*	TsCAT1	492	4	3	58–79; 227–239; 462–486	1	296–315
	TsCAT2	492	4	3	58–79; 207–229; 462–486	1	296–315
	TsCAT3	492	4	3	58–79; 207–229; 462–486	1	296–315
	TsCAT4	494	4	3	58–79; 207–229; 341–360	1	296–315
	TsCAT5	490	4	3	58–79; 207–229; 337–357	1	296–315
	TsCAT6	492	3	2	58–79; 207–229	1	296–315
	TsCAT7	492	3	2	58–79; 207–229	1	296–315
	TsCAT8	492	3	2	58–79; 207–229	1	296–315

## Data Availability

The data generated and analyzed during this study are included in this article.

## Supplementary Materials

The following supporting information can be downloaded at: https://www.mdpi.com/article/10.3390/plants13010011/s1. Table S1. Identification of disordered regions in CAT proteins using the IUPRED2A database (https://iupred2a.elte.hu/, accessed on 3 July 2023). Table S2. Sequences of the different primers used in the QRT-PCR analyses. Figure S1: Sequence alignment of CAT proteins identified in *T. dicoccoides* showing sequence conservation of those proteins. (TRIDC4BG054740.1, TRIDC7AG076360.6, TRIDC7BG073240.1, TRIDC6BG007200.1, TRIDC6BG007200.2, TRIDC6AG004940.1, TRIDC6AG004940.2). Figure S2. Multiple alignment of CAT protein sequences identified from three *Triticum* species (*T. dicocoide T. urartu* and *T. spelta*) using the cluster algorithm. Different domains are highlighted, such as one catalase core domain (PF00199 Catalase underlined in red) and one catalase immune-responsive domain (catalase-related PF06628, underlined in blue); conserved histidine (H65) and tyrosine (Y350) residues are marked with a star. *T. urartu:* (TuG1812G0700005868.01.T01, TuG1812G0700005870.01.T01, TuG1812G0700005318.01.T01, TuG1812G0600000378.01.T01); *T. spelta:* (TraesTSP4B01G347300.1, TraesTSP4D01G343400.1, TraesTSP5A01G526000.1, TraesTSP6A01G043000.1, TraesTSP6B01G059900.1, TraesTSP7A01G595800.1, TraesTSP7B01G508100.1, TraesTSP7D01G591500.1) *T. Dicoccoides* (TRIDC4BG054740.1, TRIDC7AG076360.6, TRIDC7BG073240.1, TRIDC6BG007200.1, TRIDC6BG007200.2, TRIDC6AG004940.1, TRIDC6AG004940.2). Figure S3. The phylogenetic tree of CAT proteins identified in *T. aestivum, T. turgidum, T. diccocoide, T. urartu, and T. spelta* was constructed with test maximum likehood with 1000 bootstraps by MEGA 11 and visualized by iTOL. (*T. aestivum* (TaCAT1-B: TraesCS4B02G325800; TaCAT1-D: TraesCS4D02G322700; TaCAT1-A: TraesCS5A02G498000; TaCAT2-A: TraesCS6A02G04170; TaCAT2-B: TraesCS6B02G056800; TaCAT2-D: TraesCS6D02G048300; TaCAT3-A1: TraesCS7A02G549800; TaCAT3-A2: TraesCS7A02G549900; TaCAT3-B: TraesCS7B02G473400; TaCAT3-U: TraesCSU02G105300; *T. turgidum* ssp durum (TdCAT1 WDD45561.1; TdCAT2 VAI41949.1; TdCAT3 VAI53367.1; TdCAT4 VAI53366.1; TdCAT5 VAI10245.1; TdCAT6 VAI53365.1); *T. dicocoides*: (TRIDC4BG054740.1, TRIDC7AG076360.6, TRIDC7BG073240.1, TRIDC6BG007200.1, TRIDC6BG007200.2, TRIDC6AG004940.1, TRIDC6AG004940.2); *T. urartu*: TuG1812G0700005868.01.T01, TuG1812G0700005870.01.T01, TuG1812G0700005318.01.T01, TuG1812G0600000378.01.T01; *T. spelta*: TraesTSP4B01G347300.1, TraesTSP4D01G343400.1, TraesTSP5A01G526000.1, TraesTSP6A01G043000.1, TraesTSP6B01G059900.1, TraesTSP7A01G595800.1, TraesTSP7B01G508100.1, TraesTSP7D01G591500.1. Figure S4. *TuCAT1* gene expression analysis under stressful conditions. Error bars represent standard deviation. Letters indicate significant differences (two-way ANOVA test with Tukey’s pairwise comparison). Figure S5. *TsCAT1* genes expression analysis under stressful conditions. Error bars represent standard deviation. Letters indicate significant differences (two-way ANOVA test with Tukey’s pairwise comparison). Figure S6. *TuCAT4* genes expression analysis under stressful conditions. Error bars represent standard deviation. Letters indicate significant differences (two-way ANOVA test with Tukey’s pairwise comparison). Figure S7. *TsCAT4* genes expression analysis under stressful conditions. Error bars represent standard deviation. Letters indicate significant differences (two-way ANOVA test with Tukey’s pairwise comparison).
