# Supplementary material for: Genome-Wide Identification and Expression Analysis of Catalase Gene Families in Triticeae
Source: Plants (Basel). 2023 Dec 19;13(1):11. doi: 10.3390/plants13010011 (PMC10781083; doi:10.3390/plants13010011)

Supplemental Table S1: Identification of disordered regions in CAT proteins using IUPRED2A database (https://iupred2a.elte.hu/, accessed on 03 July 2023).

		Protein length	Presence of disordered region	N-terminal	C-terminal	Percentage of disordered regions (%)	Percentage of phosphorylated sites
Triticum dicoccoide	TdcCAT1	559	2 domains	0-100	475-500	22.36	9.12 (51 residues)
	TdcCAT2	487	2 domains	0-60	475-500	17.45	7.39 (36 residues)
	TdcCAT3	492	1 domain	0-30	--	6.09	7.31 (36 residues)
	TdcCAT4	494	2 domains	10-30	405-430	9.14	7.08 (35 residues)
	TdcCAT5	494	2 domains	10-30	405-430	9.14	7.08 (35 residues)
	TdcCAT6	494	2 domains	10-30	405-430	9.14	7.08 (35 residues)
	TdcCAT7	494	2 domains	10-30	405-430	9.14	7.08 (35 residues)
Triticum urartu	TuCAT1	492	1 domain	0-30	--	6.09	7.72 (38 residues)
	TuCAT2	492	1 domain	0-30	--	6.09	7.72 (38 residues)
	TuCAT3	492	1 domain	0-30	--	6.09	7.72 (38 residues)
	TuCAT4	494	2 domains	4-25	400-438	11.94	7.48 (37 residues)
Triticum spelta	TsCAT1	492	1 domain	--	405-440	7.11	8.13 (40 residues)
	TsCAT2	492	1 domain	--	405-440	7.11	7.72 (38 residues)
	TsCAT3	492	1 domain	--	405-440	7.11	7.92 (39 residues)
	TsCAT4	494	2 domains	10-30	402-425	8.70	7.48 (39 residues)
	TsCAT5	490	2 domains	10-30	402-425	8.77	7.14 (35 residues)
	TsCAT6	492	1 domain	0-30	--	6.09	7.52 (37 residues)
	TsCAT7	492	1 domain	0-30	--	6.09	7.31 (36 residues)
	TsCAT8	492	1 domain	0-30	--	6.09	7.92 (39 residues)

Supplemental Table S2: Sequences of the different primers used in the QRT-PCR analyses.

Primer			Sequence
α-Tubulin		Tuba 1-3_Fw:	5′- GCGCCTCTCGGTTGATTAC-3′
		Tuba 1-3_Rv:	5′-GGTTTTGATGGTTGCGACT-3′
Actin		Actin_Fw:	5′-TCCCTCAGCACATTCCAGCAGAT-3′
		Actin_Rev:	5′-AACGATTCCTGGACCTGCCTCATC-3
Catalase 1	*T. urartu*	TuCAT1 Fr:	5'-CAGAAAATGAACAACTT-3'
		TuCAT1 Rv:	5'-TTCACAGGAAGCATCCAG-3'
	T. spelta	TsCAT1 Fr:	5'-CGGAGAACCGAGCAGCTG-3'
		TsCAT1 Rv:	5'-TTGGCCGGCAGCAGC-3'
	T. dicoccoides	TdcCAT1 Fr:	5'-AGTGTGTCGGGCCGATG-3'
		TdcCAT1 Rv:	5'-ACCACTATCTTGATAA-3'
Catalase 4	*T. urartu*	TuCAT4 Fr:	5'-CCACCACCCCGAGAGCCTCC-3'
		TuCAT4 Rv:	5'-GCCAGTGGAACTTGACG-3'

	T. spelta	TsCAT4 Fr:	5'-TTGGTGTCGTCGAGC-3'
		TsCAT4 Rv:	5'-CGCTCGTCGGCGGCAAG-3'
	T. dicoccoides	TdcCAT4 Fr:	5'-ATGAAGTTCATGGCGCCGTC-3'
		TdcCAT4 Rv:	5'-CCGACGACAAGATGCTGCAG-3′

Supplemental Figure S1

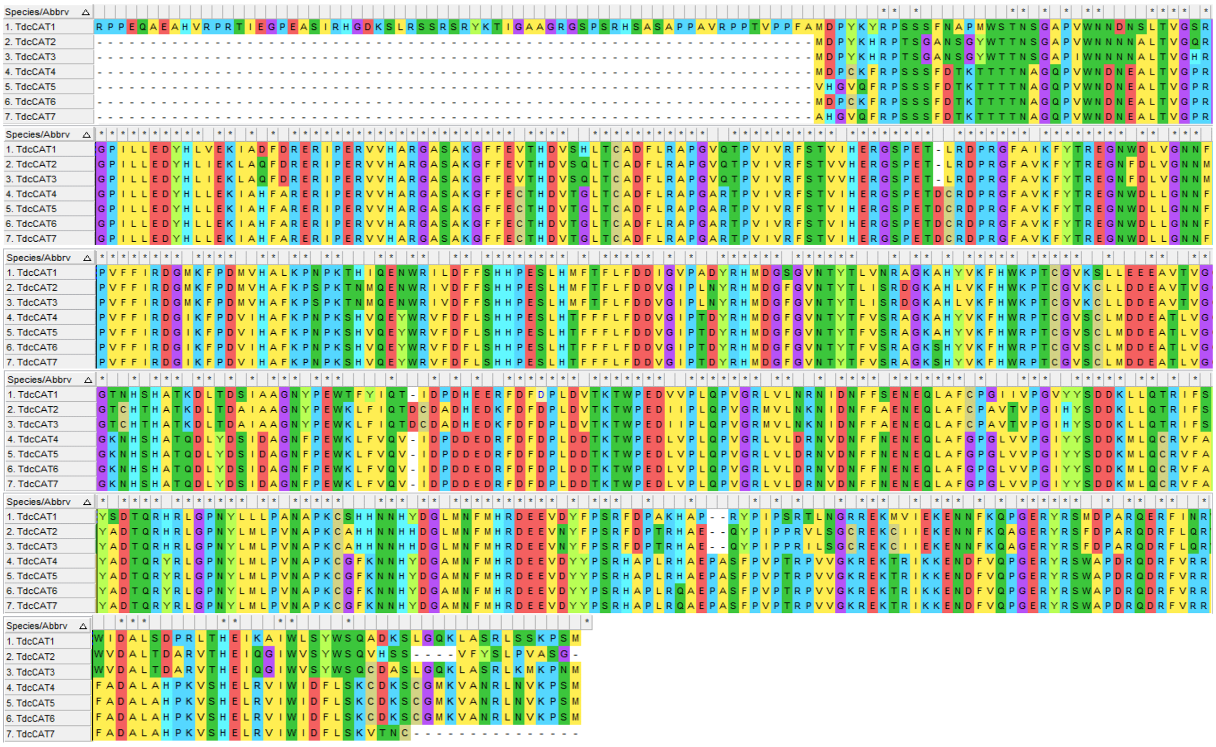

Supplemental Figure S2

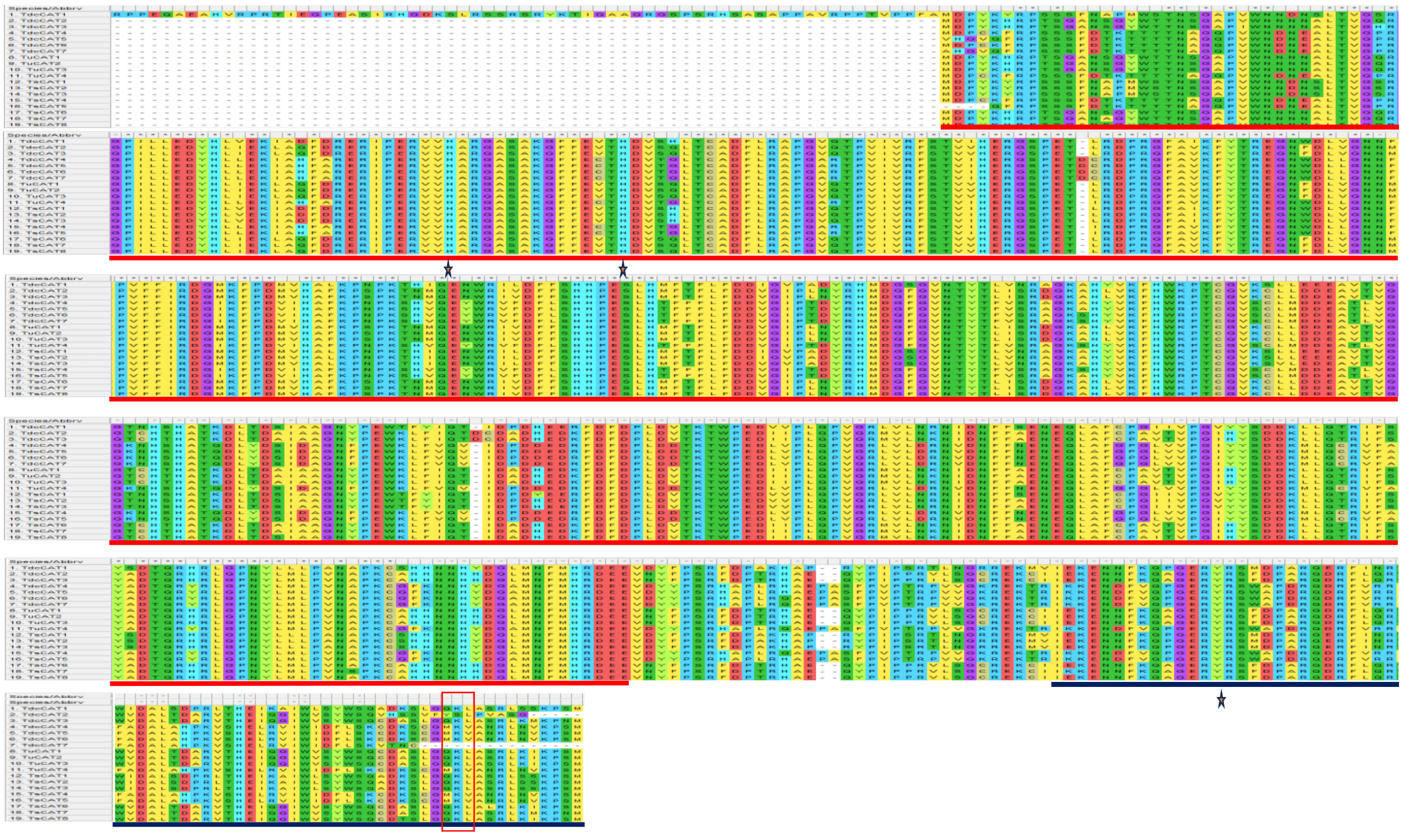

Supplemental Figure S3

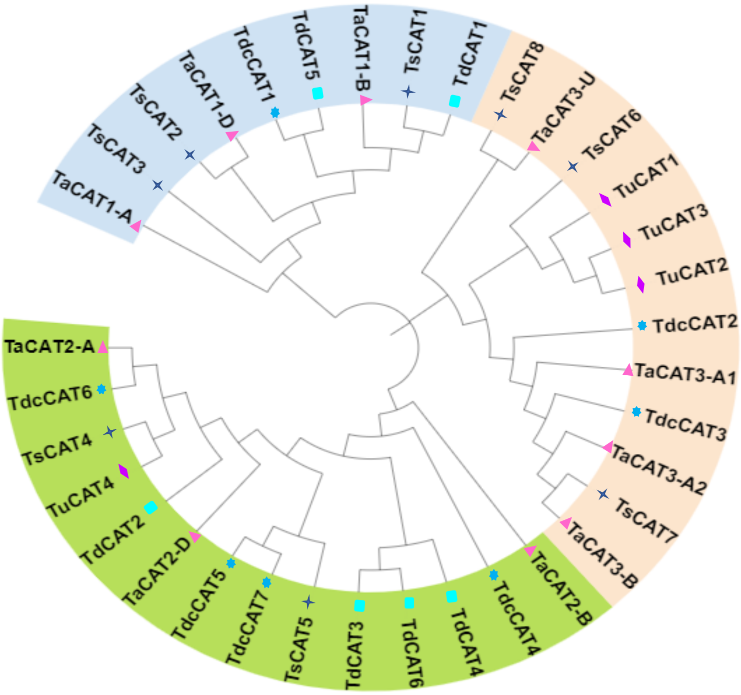

Supplemental Figure S4

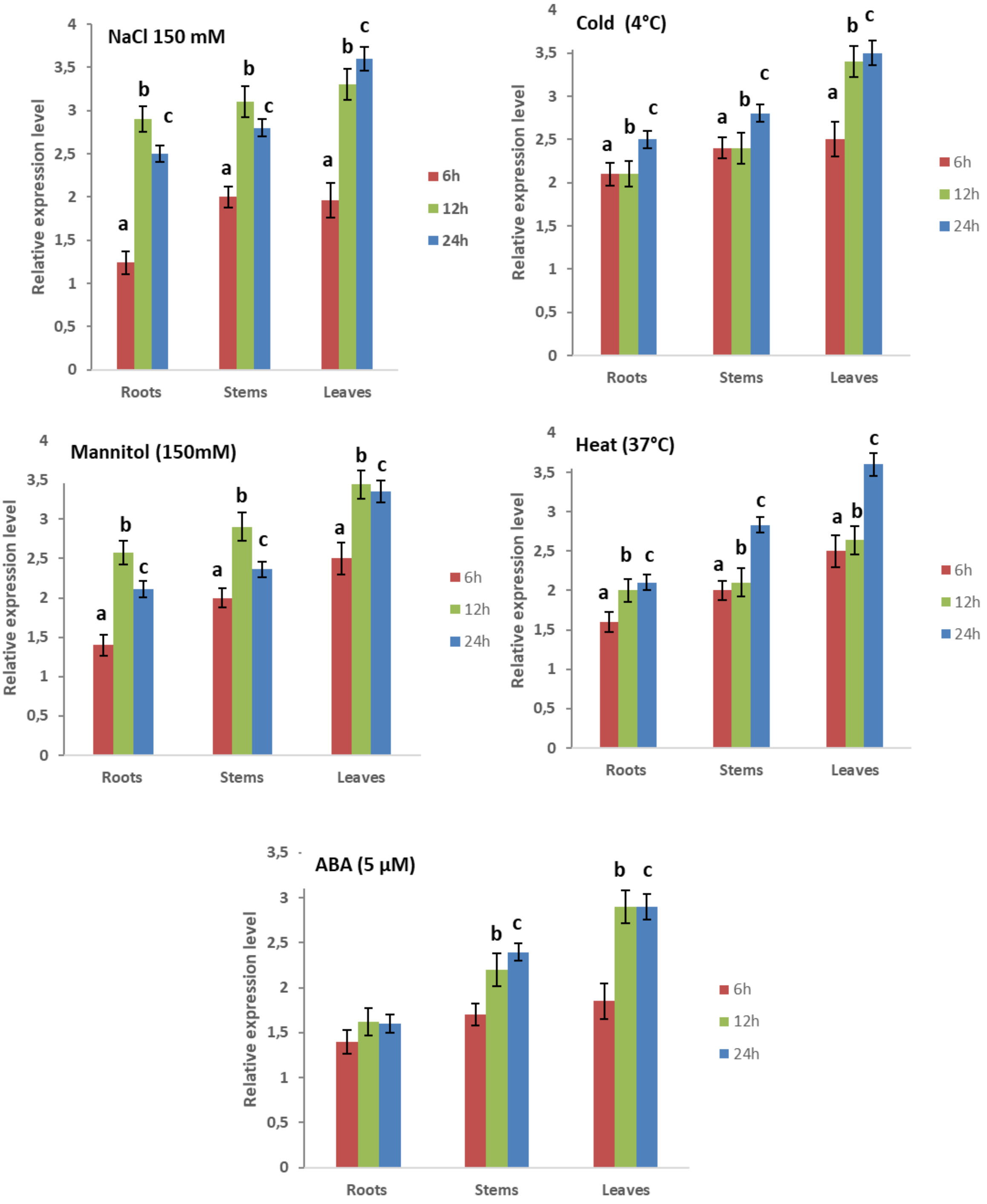

Supplemental Figure S5

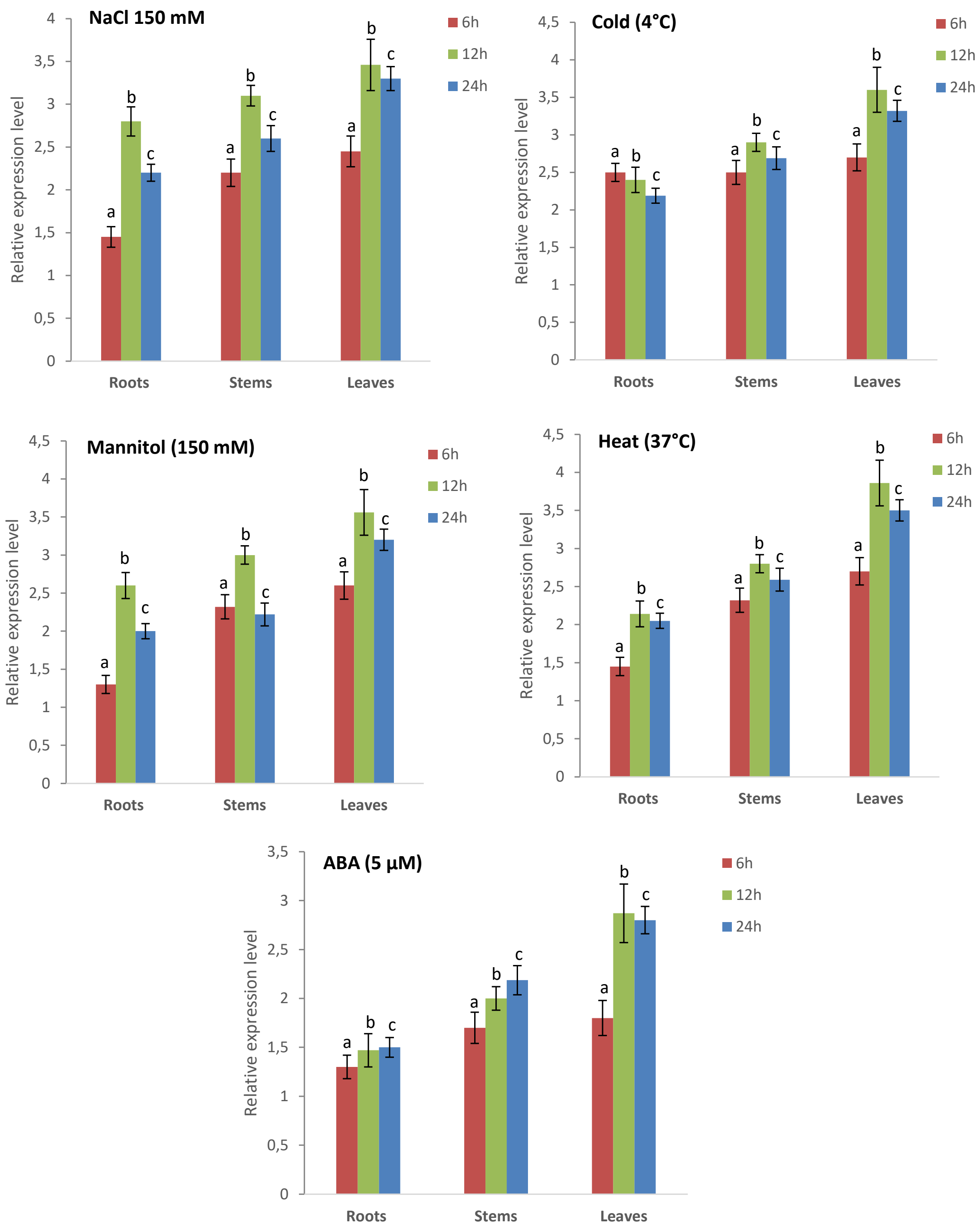

Supplemental Figure S6

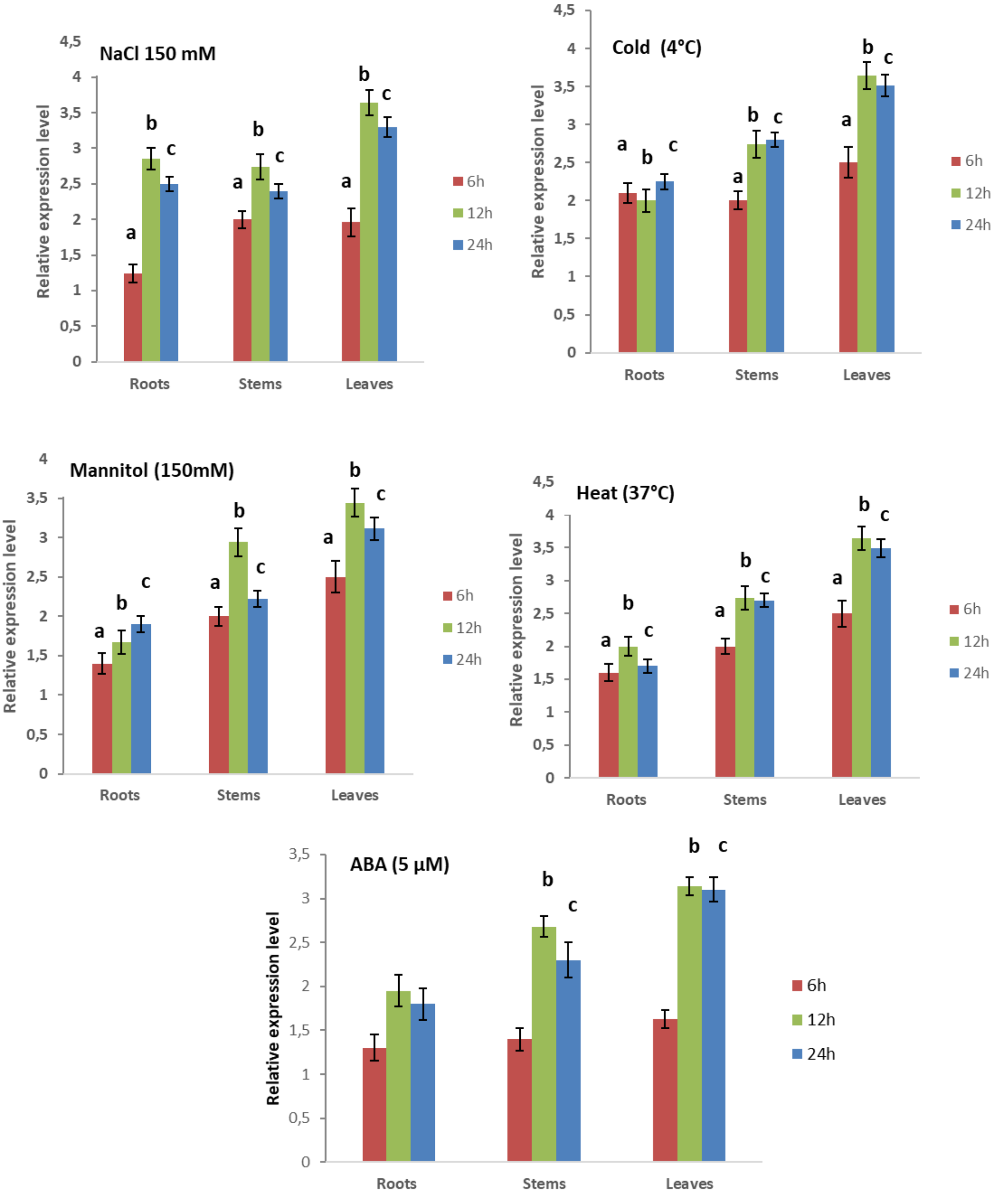

Supplemental Figure S7

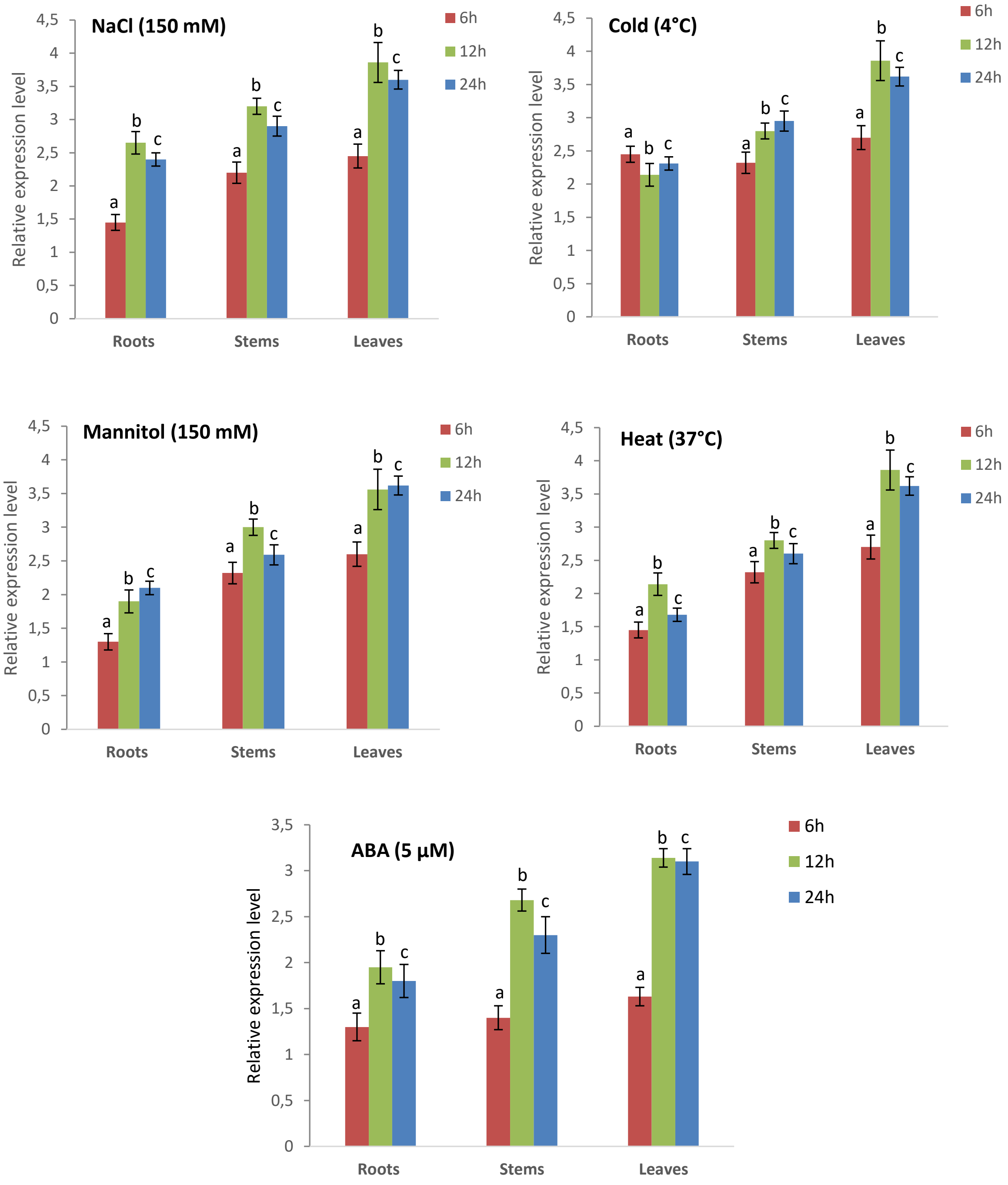

Supplement: Supplementary file 1 [file plants-13-00011-s001.zip › plants-2696807-supplementary.pdf]
